# Deep Learning-Based Target Tracking and Classification for Low Quality Videos Using Coded Aperture Cameras

**DOI:** 10.3390/s19173702

**Published:** 2019-08-26

**Authors:** Chiman Kwan, Bryan Chou, Jonathan Yang, Akshay Rangamani, Trac Tran, Jack Zhang, Ralph Etienne-Cummings

**Affiliations:** 1 Applied Research LLC, Rockville, MD 20850, USA; 2 Google, Inc., Mountain View, CA 94043, USA; 3 Department of Electrical and Computer Engineering, Johns Hopkins University, Baltimore, MD 21218, USA; 4 Department of Brain and Cognitive Sciences, MIT, Cambridge, MA 02138, USA

**Keywords:** compressive sensing, pixel-wise code exposure camera, YOLO, ResNet, target tracking, target classification, optical, MWIR

## Abstract

Compressive sensing has seen many applications in recent years. One type of compressive sensing device is the Pixel-wise Code Exposure (PCE) camera, which has low power consumption and individual control of pixel exposure time. In order to use PCE cameras for practical applications, a time consuming and lossy process is needed to reconstruct the original frames. In this paper, we present a deep learning approach that directly performs target tracking and classification in the compressive measurement domain without any frame reconstruction. In particular, we propose to apply You Only Look Once (YOLO) to detect and track targets in the frames and we propose to apply Residual Network (ResNet) for classification. Extensive simulations using low quality optical and mid-wave infrared (MWIR) videos in the SENSIAC database demonstrated the efficacy of our proposed approach.

## 1. Introduction

Compressive measurements [1] can save data storage and transmission costs. The measurements are normally collected by multiplying the original vectorized image with a Gaussian random matrix. Each measurement is a scalar and the measurement is repeated many times. The saving is achieved because the number of measurements is much fewer than the number of pixels in the original frame. To track a target using compressive measurements, it is required to reconstruct the image scene.

However, it is difficult, if not impossible, to carry out target tracking and classification directly using the compressive measurements that are generated by the Gaussian random matrix. This is because the target location, and target size and shape information in an image frame is destroyed by the Gaussian random matrix.

Recently, a new compressive sensing device known as Pixel-wise Code Exposure (PCE) camera was proposed [2]. In [2], the original frames were reconstructed using *L*_1_ [3] or *L*_0_ [4,5,6] sparsity based algorithms. It is well-known that it is computationally intensive to reconstruct the original frames and hence real-time applications may be infeasible. Moreover, information may be lost in the reconstruction process [7]. For real-time applications, it will be important to carry out target tracking and classification using compressive measurement directly. Although there are some tracking papers [8] in the literature that appear to be using compressive measurements, they are actually still using the original video frames for tracking.

In this paper, we propose a target tracking and classification approach in compressive measurement domain for long range and low quality optical and MWIR videos. First, YOLO [9] is used for target tracking. The training of YOLO requires image frames with known target locations, which can be easily done. It should be noted that YOLO does have a built-in classifier. However, its performance is not good based on our past experience [10,11,12,13,14]. As a result, ResNet [15] has been used for classification because some customized training can be done via data augmentation of the limited video frames. Although other deep learning based classifiers could be used, we chose ResNet simply because its ability to avoid saturation issues. Our proposed approach was demonstrated using low quality videos (long range, low spatial resolution, and poor illumination) in the SENSIAC database. The tracking and classification results are reasonable up to certain ranges. Big improvement has been noticed over conventional trackers [16,17]. Moreover, conventional trackers do not work well for multiple targets [10].

Although the proposed approach has been applied to shortwave infrared (SWIR) videos in an earlier paper [10], the application of the proposed approach to SENSIAC videos is completely new. Most importantly, the video quality in terms of spatial resolution and illumination in SENSIAC videos is much worse than those SWIR videos in [10]. The SENSIAC database contains both optical and MWIR videos collected from ranges of 1000 m up to 5000 m. In some videos, cameras also move and there are also air turbulence caused by desert heat. Some dust caused by moving vehicles can be seen in some optical videos. There are seven types of vehicles, which are hard to distinguish from long ranges. For MWIR videos, there are daytime and nighttime videos as well. We have demonstrated that the proposed deep learning approach is general and applicable to low quality optical and MWIR videos. Our studies also showed that optical has better tracking and classification performance than MWIR daytime videos and MWIR videos are more appropriate for nighttime operations.

It is worth to briefly review some state-of-the-art algorithms that performs action inference or object classification directly using compressive measurements. We will also highlight the differences between our approach and those other approaches.

Paper [18] presents a reconstruction-free approach to action inference. The key idea is to build smashed filters using training samples that are affine transformed to a canonical viewpoint. The approach works very well even for 100 to 1 compression. However, the approach is for action inference (e.g., a moving car or some other actions), not for target detection, tracking, and classification (e.g., the moving car is a Ram, not a Jeep) in compressed measurement domain. Moreover, the smashed filter may assume that the camera is stationary and the angle is fixed. Extending the approach to target tracking and classification with moving cameras may be non-trivial.

In [19], a CNN approach was presented to perform image classification directly in compressed measurement domain. The input image is assumed to be cropped and centered, and there is only one target in each image. This is totally different from our paper in which the target can be anywhere in the image frames.

Papers [20,21] are similar in spirit to [19]. Both papers discussed direct object classification using compressed measurement. However, both papers assumed that the targets/objects are already centered. Moreover, it is a classification study only without target detection and tracking. This is similar to the ResNet portion of our approach. Again, the problem and scenarios in these papers are different from ours because the target can be anywhere in the video frames in our paper.

Strictly speaking, the approach in [22] is not reconstruction free. The integral image is one type of reconstructed image. After the integral image is obtained, other tracking filters are then applied. There was also no discussion of object classification. Our paper does not require any image reconstruction.

Reference [23] is interesting in that a random mask is applied to conceal the actual contents of the original video. They call the video with random mask a coded aperture video. If one looks closely, the coded aperture idea in [23] is very different from the PCE idea in our paper. In addition, the key idea in [23] is about action recognition (similar to [18]), not object tracking and classification. Extending the idea in [23] to object tracking and classification may not be an easy task.

Reference [24] presents an object detection approach using correlation filters and sparse representation. There was no object classification. No reconstruction of compressive measurements is needed. The results are quite good. One potential limitation of the idea in [24] is that the sparsity approach may be very time consuming when the dictionary size is large and hence may not be suitable for near real-time applications. Different from [24], our paper focuses on object detection, tracking, and classification. Once trained, our approach can work in a near real-time fashion.

In [25], the authors present an approach to extracting features out of the compressed measurements and then uses the features to create a proxy image, which is then used for action recognition. If our interpretation is correct, this approach may not be considered as a reconstruction free approach because there is a construction of a proxy image. Similar to [19,20,21], it appears the approach is suitable for stationary camera cases and also the objects are already centered in the images. In our approach, the camera can be non-stationary and targets can be anywhere in the image.

Paper [26] presents an online reconstruction free approach to object classification using compressed measurements. Similar to [19,20,21,25], the approach assumes the object is already at the center of the image. For an image frame where the target location is unknown, then it is not clear on how this approach can be applied to handle the above situation. We faced the same problem two years ago when we investigated a sparsity based approach [7] that directly classifies objects using compressive measurements. However, we still could not solve the classification issue in which the target is located in a small and random location of an image frame. The methods in [19,20,21,25,26] also did not address the above mentioned issue.

This paper is organized as follows: in Section 2, we describe some background materials, including the PCE camera, YOLO, ResNet, SENSIAC videos, and performance metrics. In Section 3, we present some tracking results using a conventional tracker, which clearly has poor performance when using compressive measurements directly. Section 4, Section 5 and Section 6 then focus on presenting the deep learning results. In particular, Section 4 summarizes the tracking and classification results using optical videos. Section 5 and Section 6 summarize the tracking and classification results for MWIR daytime and nighttime videos, respectively. Finally, we conclude our paper with some remarks for future research. To make our paper easier to read, we have moved some tracking and classification results to the Appendices.

## 2. Materials and Methods

### 2.1. PCE Imaging and Coded Aperture

**Figure 1:**
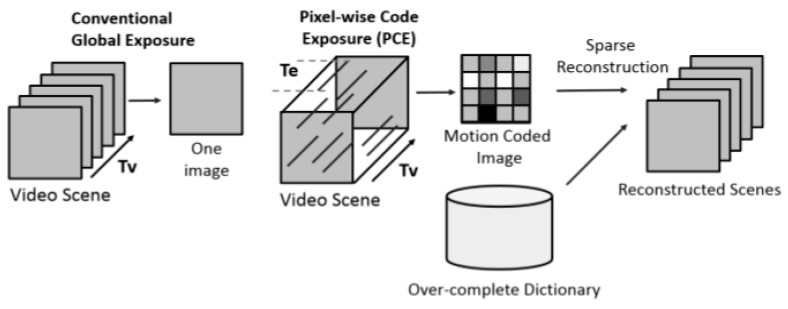
Conventional camera vs. Pixel-wise Coded Exposure (PCE) Compressed Image/Video Sensor [2].

Here, we briefly review the PCE or Coded Aperture (CA) video frames [2]. The differences between a conventional video sensing scheme and PCE are shown in Figure 1. First, conventional cameras capture frames at 30 or 5 or some other frames per second. A PCE camera, however, captures a compressed frame called motion coded image over a fixed period of time (*T_v_*). For instance, it is possible to compress 20 original frames into a single motion coded frame. The compression ratio is very significant. Second, the PCE camera allows one to use different exposure times for different pixel locations. Consequently, high dynamic range can be achieved. Moreover, power can also be saved via low sampling rate. One notable disadvantage of PCE is that, as shown in the right-hand side of Figure 1, an over-complete dictionary is needed to reconstruct the original frames and this process may be very computationally intensive and may prohibit real-time applications.

The coded aperture image $Y\in R^{M\times N}$ is obtained by:

(1) $$Y(m,n)=\sum_{t=1}^{T}S(m,n,t)\cdot X(m,n,t)$$

where $X\in R^{M\times N\times T}$ contains a video scene with an image size of *M* × *N* and the number of frames of *T*; $S\in R^{M\times N\times T}$ contains the sensing data cube, which contains the exposure times for pixel located at (*m*, *n*, *t*). The value of *S* (*m*, *n*, *t*) is 1 for frames *t* ∈ [*t*_start_, *t*_end_] and 0 otherwise. [*t*_start_, *t*_end_] denotes the start and end frame numbers for a particular pixel.

The video scene $X\in R^{M\times N\times T}$ can be reconstructed via sparsity methods (*L*_1_ or *L*_0_). Details can be found in [2]. However, the reconstruction process is time consuming and hence not suitable for real-time applications.

Instead of performing sparse reconstruction on PCE images, our scheme directly works on the PCE images. Utilizing raw PCE measurements has several challenges. First, moving targets may be smeared if the exposure times are long. Second, there are also missing pixels in the raw measurements because not all pixels are activated during the data collection process. Third, there are much fewer frames in the raw video because a number of original frames are compressed into a single coded frame. This means that the training data will be limited.

In this paper, we have focused on simulating PCE measurement. We then proceed to demonstrate that detecting, tracking, and classifying moving objects is feasible. We carried out multiple experiments with three diverse sensing models: PCE/CA Full, PCE/CA 50%, and PCE/CA 25%.

The PCE Full Model (PCE Full or CA Full) is quite similar to a conventional video sensor: every pixel in the spatial scene is exposed for exactly the same duration of one second. This simple model still produces a compression ratio of 30:1. The number “30” is a design parameter. Based on our sponsor’s requirements, in our experiments, we have used 5 frames, which achieved 5 to 1 compression already.

Next, in the sensing model labeled as PCE 50% or CA 50%, there are roughly 1.85% pixels being activated in each frame with an exposure time of *T_e_* = 133.3 ms. Since we are summing up 30 frames into a single coded frame, summing 30 frames of 1.85% is equivalent to 55.5% of all pixels that have exposure in the coded frame. Because the pixels are randomly selected in each frame, some pixels may overlap. So, activating 1.85% in each frame is roughly equivalent to 50% of activated pixels in the coded frame. Similarly, for PCE 25 case, the percentage of activated pixels in each frame will be reduced by half from 1.85% to 0.92%. The exposure duration is still set at the same conventional 4-frame duration. Table 1 below summarizes the comparison between the three sensing models for data and power savaging ratios. Details can be found in [10].

### 2.2. YOLO Tracker

YOLO [9] is fast and similar to Faster R-CNN [27]. We picked YOLO rather than Faster R-CNN simply because of easier installation and compatibility with our hardware. The training of YOLO is quite simple, as only images with ground truth target locations are needed.

YOLO is mainly performing object detection. The tracking is achieved by detection. That is, the detected object locations from all frames are connected together to form object tracks. Conventional trackers usually require a human operator to manually put a bounding box on the target in the first frame. This is not only inconvenient, but also may not be practical, especially for long term tracking where tracking may need to be re-started after some frames. Comparing with conventional trackers [16,17], YOLO does not require any information on the initial bounding boxes. Moreover, YOLO can handle multiple targets simultaneously.

**Figure 2:**
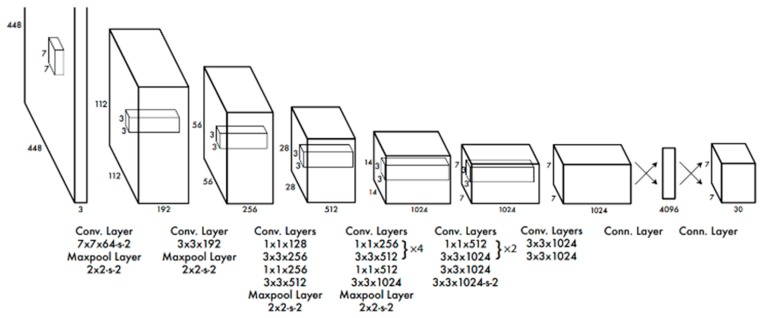
24 convolutional layers followed by 2 fully connected layers for YOLO version 1 [9].

YOLO also comes with a classification module. However, based on our evaluations, the classification accuracy using YOLO is not good as can be seen in [10,11,12,13,14]. For completeness, we include a block diagram of YOLO-version 1 [9] in Figure 2. The input image needs to be resized to 448 × 448. There are 24 layers. YOLO version 2 has been used in our experiments.

### 2.3. ResNet Classifier

**Figure 3:**
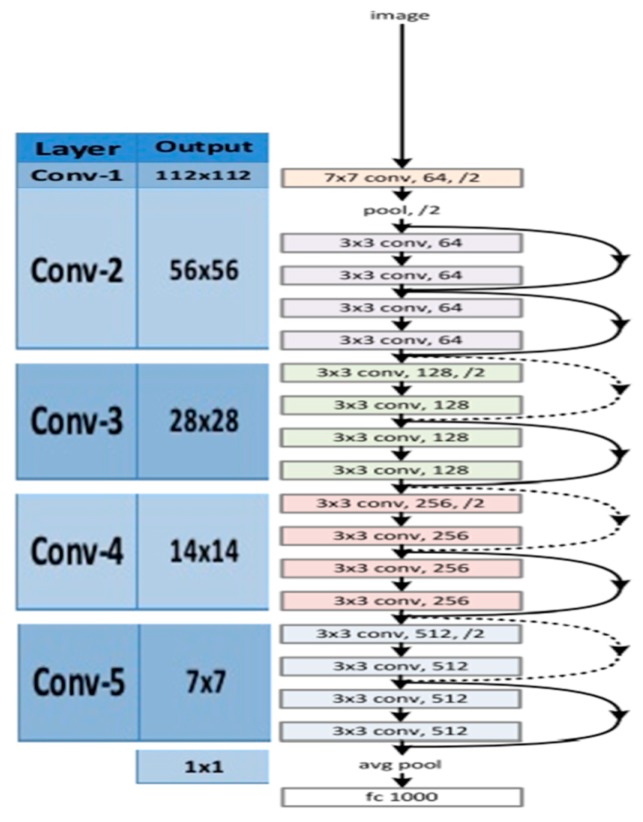
Architecture of ResNet-18. Figure from [15].

A common problem in deep CNN is performance saturation. The ResNet-18 model is an 18-layer convolutional neural network (CNN), which avoids performance saturation in training deeper layers. The key idea in ResNet-18 model is an identity shortcut connection, which skips one or more layers. Figure 3 shows the architecture of an 18-layer ResNet.

Training of ResNet requires target patches. The targets are cropped from training videos. Mirror images are then created. We then perform data augmentation using scaling (larger and smaller), rotation (every 45 degrees), and illumination (brighter and dimmer) to create more training data. For each cropped target, we are able to create a data set with 64 more images.

The relationship between YOLO and ResNet is that YOLO determines where the targets are and bounding boxes are put around the targets. The pixels inside the bounding boxes will be fed into the ResNet-18 for classification.

The training of ResNet was done as follows: first, the targets are cropped from training videos at a particular range in the SENSIAC database. Second, mirror images were then generated. Third, we then applied data augmentation using scaling (larger and smaller), rotation (every 45 degrees), and illumination (brighter and dimmer) to generate more training data. For every cropped target, 64 additional synthetic targets were generated.

### 2.4. Data

**Figure 4:**
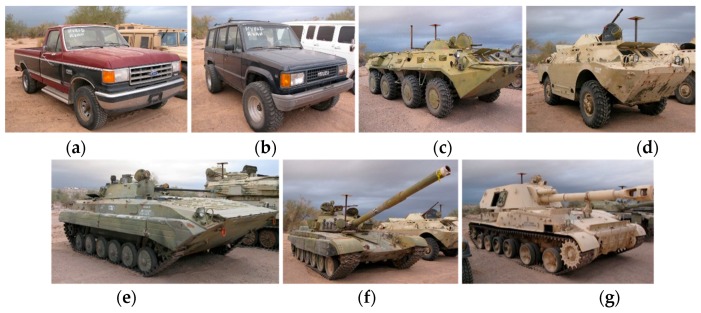
Seven targets in SENSIAC: (**a**) Truck; (**b**) SUV; (**c**) BTR70; (**d**) BRDM2; (**e**) BMP2; (**f**) T72; and (**g**) ZSU23-4.

To fulfill our sponsor’s requirements, our research objective is to perform tracking and classification of seven vehicles using the SENSIAC videos. There are optical and mid-wave infrared (MWIR) videos collected at distances ranging from 1000 to 5000 m with 500 m increments. The seven types of vehicles are shown in Figure 4. These videos are challenging for several reasons. First, the target sizes are small due to long distances. This is quite different from some benchmark datasets such as MOT Challenge [28] where the range is short and the targets are big. Second, the target orientations also change drastically. Third, the illuminations in different videos are also different. Fourth, the cameras also move in some videos. Fifth, both optical and MWIR videos are present. Sixth, some environmental factors such as air turbulence due to desert heat are also present in some optical videos.

Although there are other benchmark videos such as the MOT Challenge Database, our sponsor is aware of that database. However, since our sponsor is interested in long range, small targets (vehicles), and gray scale videos, MOT Challenge dataset does not meet the requirements of our sponsor. Most videos in the MOT Challenge dataset contain human subjects at close distance and the videos are color videos. Moreover, we have limited project funding to only focus on some relevant datasets. Consequently, we did not have time to explore other videos such as MOT Challenge.

Having said the above, we would like to mention that, in our experiments, a total of 378 videos comprising seven vehicles, six long distance ranges (1000 to 3500 m in 500 m increments), three imaging modalities (optical, MWIR daytime, MWIR nighttime), and three coded aperture modes. In short, our experiments are very comprehensive. No one has carried out such a comprehensive tracking and classification study for SENSIAC dataset in the compressed measurement domain. In this regard, our paper has reasonable contributions to the research community.

**Figure 5:**
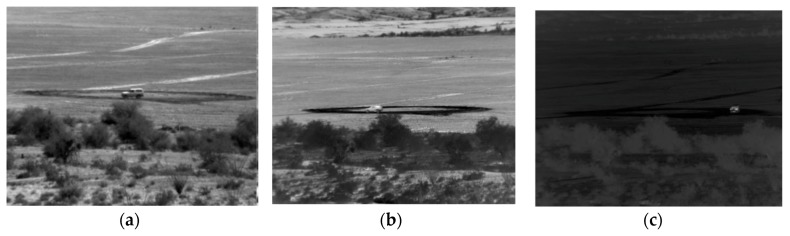
Frames from optical and MWIR videos. Although the videos were collected from roughly the same range, the vehicle sizes and characteristics are somewhat different, making the tracking and classification very difficult. Three scenarios are shown: (**a**) Optical at 1000 m; (**b**) MWIR daytime at 1000 m; and (**c**) MWIR nighttime at 1000 m.

Here, we briefly highlight the background for optical and MWIR videos. Figure 5 shows a few examples of optical and MWIR images. The optical and MWIR videos have very different characteristics. Optical imagers have a wavelength between 0.4 and 0.8 microns and MWIR imagers have a wavelength range between 3 and 5 microns. Optical cameras require external illuminations whereas MWIR counterparts do not need external illumination sources because MWIR cameras are sensitive to heat radiation from objects. Consequently, target shadows can affect the target detection performance in optical videos. However, there are no shadows in MWIR videos. Moreover, atmospheric obscurants cause much less scattering in the MWIR bands than in the optical band. As a result, MWIR cameras are tolerant of heat turbulence, smoke, dust and fog.

### 2.5. Performance Metrics

In our earlier paper [10,11,12,13,14], we have included some tracking results where conventional trackers such as GMM [17] and STAPLE [16] were used. The tracking performance was poor when there are missing data.

Although there may be other metrics that could be used, some of the metrics have similar meanings. Hence, we believe that the following popular and commonly used metrics are sufficient for evaluating the tracker performance:

- Center Location Error (CLE): It is the error between the center of the bounding box and the ground-truth bounding box.
- Distance Precision (DP): It is the percentage of frames where the centroids of detected bounding boxes are within 20 pixels of the centroid of ground-truth bounding boxes.
- EinGT: It is the percentage of the frames where the centroids of the detected bounding boxes are inside the ground-truth bounding boxes.
- Number of frames with detection: This is the total number of frames that have detection.

For classification, we used confusion matrix and classification accuracy as performance metrics.

## 3. Conventional Tracking Results

**Figure 6:**
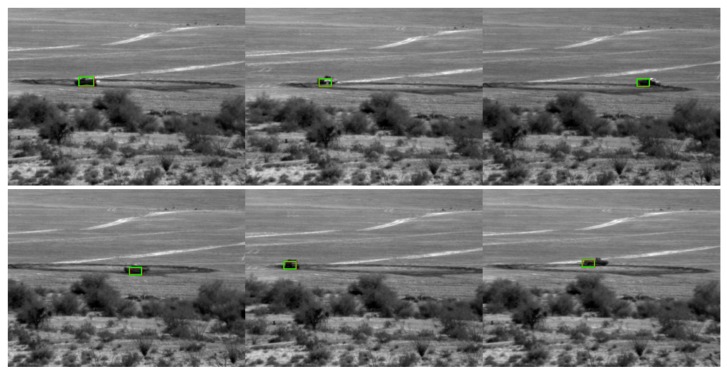
STAPLE tracking results for the PCE full case. Frames: 10, 30, 50, 70, 90, and 110 are shown here.

**Figure 7:**
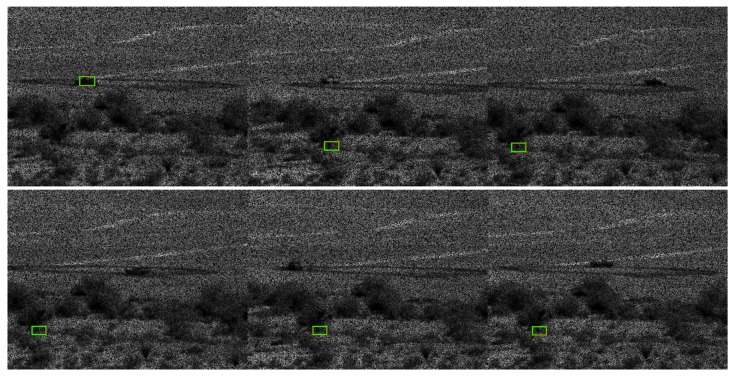
STAPLE tracking results for the PCE 50% case. Frames: 10, 30, 50, 70, 90, and 110 are shown here. The green boxes are not on targets.

**Figure 8:**
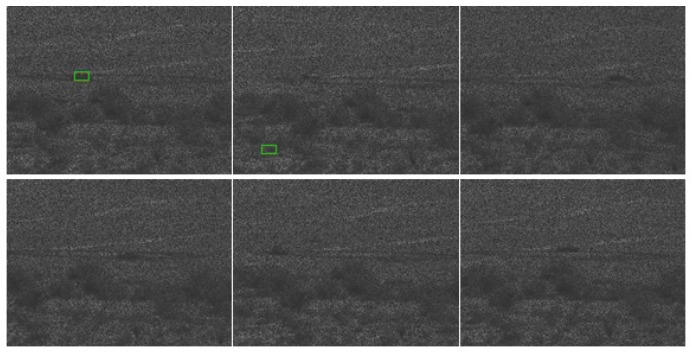
STAPLE tracking results for the PCE 25% case. Frames: 10, 30, 50, 70, 90, and 110 are shown here. Many frames do not have detections. The bounding boxes completely miss the targets.

We first present some tracking results for optical videos at a range of 1000 m using a conventional tracker known as STAPLE [16]. The compressive measurements based on the PCE principle have been obtained. Here, every five frames were compressed into one frame. STAPLE requires the target location to be known in the first frame. After that, STAPLE learns the target model online and tracks the target. However, in two of three cases (PCE 50%, and PCE 25%) as shown in Figure 6, Figure 7 and Figure 8, STAPLE was not able to track any targets in subsequent frames. This shows the difficulty of target tracking using PCE cameras. Moreover, in our earlier studies for SWIR videos [10], we already compared conventional trackers with deep learning based trackers. It was observed that conventional trackers do not work well in compressive measurement domain. We would like to mention that, it is somewhat unfair to the authors of [16] because STAPLE was not designed to handle videos in compressed measurement domain. Therefore, in our subsequent studies shown in Section 4, Section 5 and Section 6, we focused only on deep learning results because of the above observations.

## 4. Tracking and Classification Results Using SENSIAC Optical Videos

This study focuses on the case of tracking and classification using a combination of YOLO and ResNet for coded aperture cameras. The compressive measurements are simulated using PCE camera principle. There are three cases. PCE full refers the compression of 5 frames to 1 with no missing pixels. PCE 50 is the case where we compress 5 frames to 1 and at the same time, only 50% of pixels are activated for a length of 4/30 s. PCE 25 is similar to PCE 50 except that only 25% of the pixels are activated for 4/30 s.

### 4.1. Tracking

We used 1500 and 3000 m videos to train two separate YOLO models. The 1500 m model was used for 1000 to 2000 m ranges and the 3000 m model was for 2500 to 3500 m ranges. Longer range videos (4000 to 5000 m) were not used because the targets are too small.

**Figure 9:**
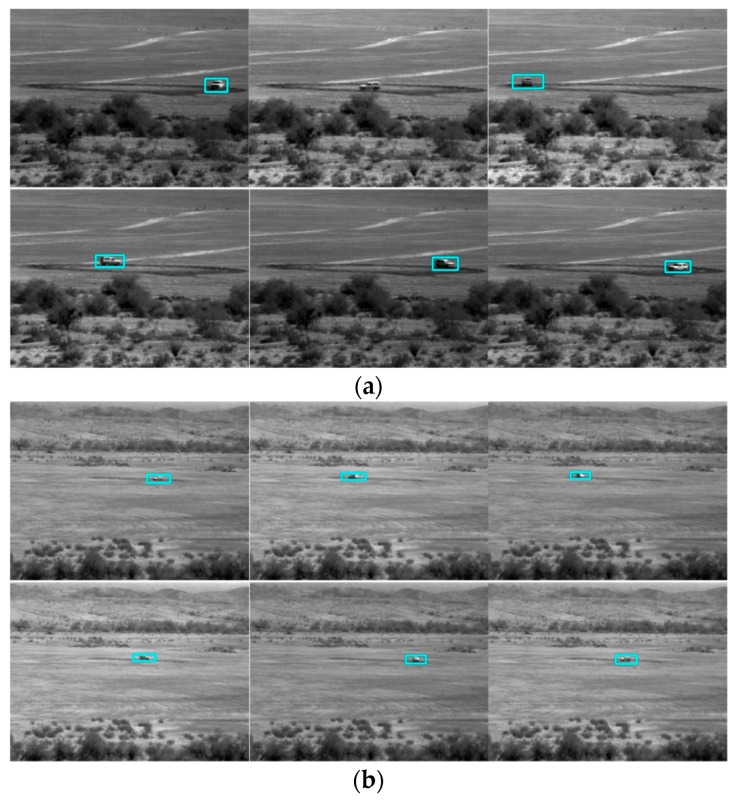

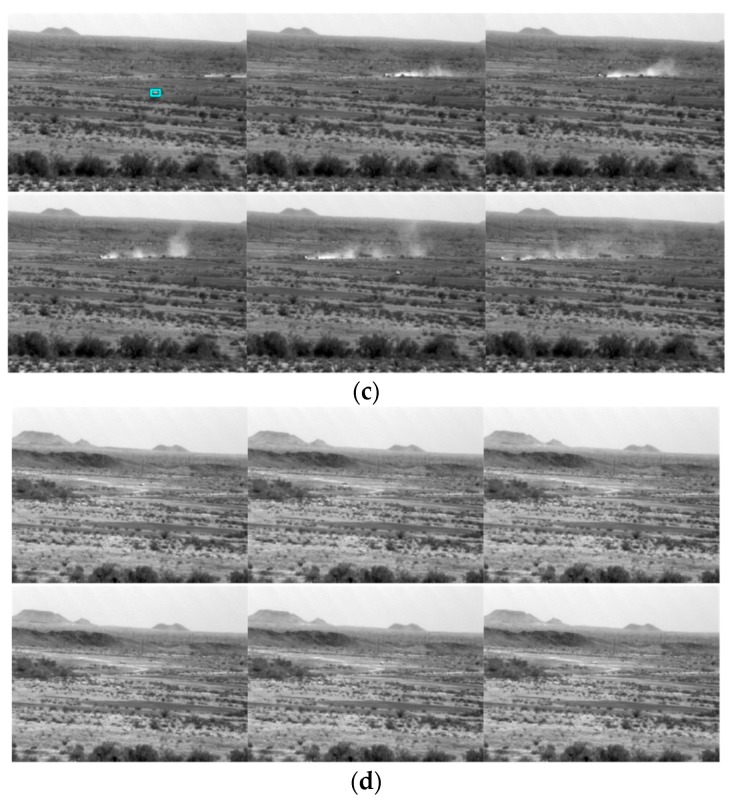
Tracking results for frames 1, 63, 126, 189, 252, and 315 in the PCE full (optical videos) case. The vehicle is SUV. Coded aperture compresses every five frames into one. (**a**) 1000 m; (**b**) 2000 m; (**c**) 2500 m; and (**d**) 3500 m.

Table 2 and two tables in Appendix A show the tracking results for PCE full, PCE 50, and PCE 25, respectively. The trend is that when image compression increases, the performance drops accordingly. Table 2 summarizes the PCE full case. The tracking performance is good up to 3000 m. For PCE 50 case (see the first table in Appendix A), the tracking is only good up to 2000 m. We also observe some poor tracking results for some vehicles (BRDM2 at 2000 m). For PCE 25 case (second table in Appendix A), the tracking is only reasonable up to 1500 m. There are also some poor detection results even for 1000 and 1500 m ranges. The above observations can be corroborated in the snapshots shown in Figure 9 and two figures in Appendix A where we can see that some targets do not have bounding boxes around them in the high compression cases. We can also observe some dusts caused by the moving vehicles. Dusts can seriously affect the tracking and classification performance. In Figure 9 (PCE full case), one can see that most of the sampled frames in 2500 and 3500 m videos do not have any detections. We did not include 1500 and 3000 m snapshots because those videos are used in the training. In the first figure (PCE 50) in Appendix A, it can be seen that the detection performance deteriorates, as most of the sampled frames do not have detections. The tracking results in second figure (PCE 25) in Appendix A are not good even for 1000 m range. The selected video contains the SUV vehicle, which unfortunately has 11% detection in the 1000 m range.

From this study alone, it is very clear to see the difficulty of target tracking using compressive measurement directly for the SENSIAC videos. Challenges mean opportunities. We hope researchers will continue along this path.

### 4.2. Classification Results

Here, we applied ResNet for classification. Two models were obtained. One used the 1500 m videos for training and then 1000 m and 2000 m videos for testing. The other one used the 3000 m videos for training and 2500 m and 3500 m videos for testing. It should be noted that classification is performed only when there is good detection results from the YOLO tracker. For some frames in the PCE 50 and PCE 25 cases, there may not be any positive detection results and, for those frames, we do not generate any classification results.

Table 3 and two tables in Appendix B show the classification results using ResNet for PCE full, PCE 50, and PCE 25 cases. In each table, the left side contains the confusion matrix and the last column contains the classification accuracy. From Table 3 (PCE full), the accuracy is reasonably good up to 1500 m range. At 2000 m range, the accuracy fluctuates a lot among the different vehicles. For ranges beyond 2500 m, the accuracy is low. From first table (PCE 50) in Appendix B, the accuracy is only good for 1500 m, which is the range that we used for training. Other ranges are not good. Similarly, the results in the second table (PCE 25) in Appendix B are all bad. This study clearly shows that it is difficult to get good classification results for SENSIAC optical videos in which the targets are small. More research is needed.

### 4.3. Summary (Optical)

We collected some statistics from Table 2, Table 3, and those tables in Appendix A and Appendix B and summarize those averages in Table 4. For optical videos, the performance of tracking and classification is good up to 2000 m in the PCE full case. For PCE 50, the tracking is still reasonable, but the classification is not good. For PCE 25, even the tracking is not very good for 1000 m range. The classification is even worse for PCE 25. More research is needed in order to get better performance.

## 5. Tracking and Classification Using MWIR Daytime Videos

The SENSIAC database contains MWIR daytime and nighttime videos. Here, we focus on daytime videos.

### 5.1. Tracking

Similar to the optical case, we trained two models. One used 1500 m videos and the other used 3000 m videos. For the 1500 m model, videos from 1000 and 2000 m videos were used for testing; for the 3000 m model, videos from 2500 and 3500 m were used for testing. Table 5 and two additional tables in Appendix C show the tracking results for PCE full, PCE 50, and PCE 25, respectively.

**Figure 10:**
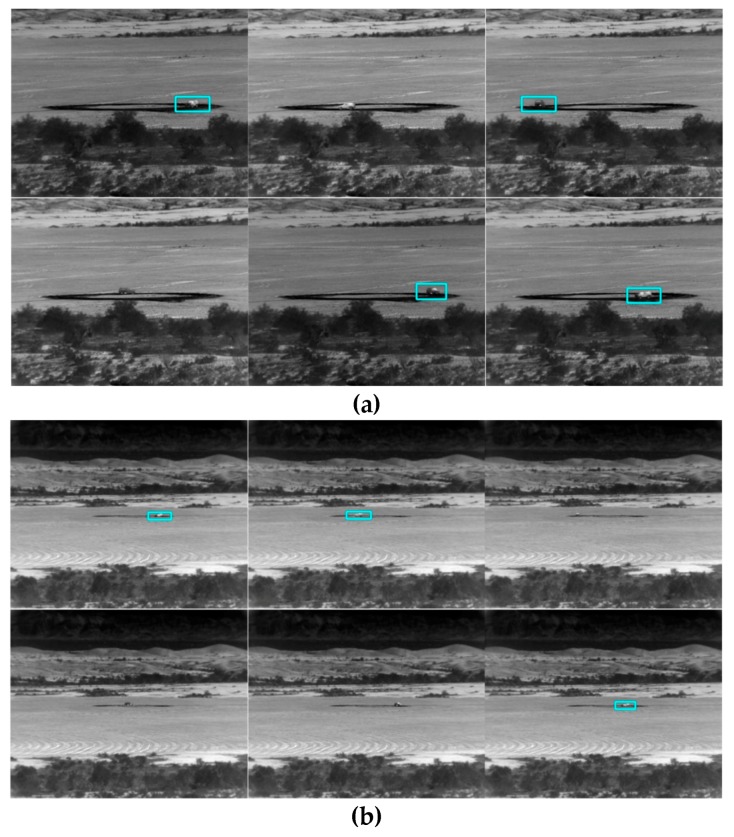

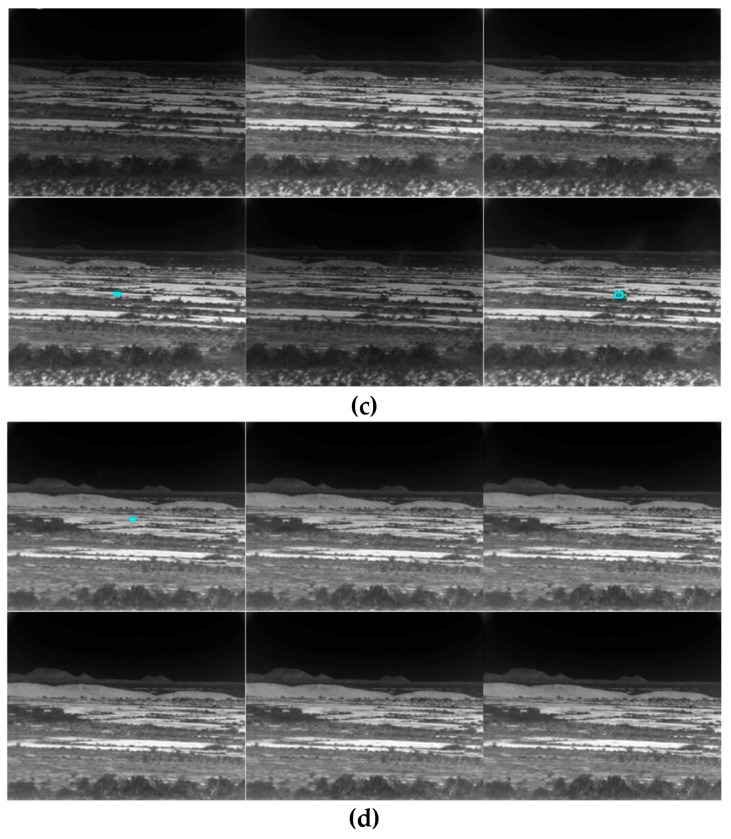
Tracking results for frames 1, 60, 119, 178, 237, and 296 for the PCE full (MWIR daytime) case. The vehicle is SUV. Only some frames have detections. (**a**) 1000 m; (**b**) 2000 m; (**c**) 2500 m; and (**d**) 3500 m.

From Table 5 (PCE full), the tracking results for 1000 to 2500 m are reasonable. Some vehicles have better numbers than others. From the table for PCE 50 in Appendix C, the performance deteriorates drastically. Even for the 1500 and 3000 m ranges, the results are not good. From the table for PCE 25 in Appendix C, the performance gets even worse. This can be confirmed in the snapshots shown in Figure 10 and two additional figures in Appendix C where we can see that some targets do not have bounding boxes around them in the high compression cases. An observation is that the tracking performance in MWIR daytime videos is generally worse than that of using optical videos.

### 5.2. Classification (MWIR Daytime)

Similar to the optical case, we trained two ResNet classifiers: one for the 1500 m range and another for the 3000 m range. For the 1500 and 3000 m models, videos from 1000 and 2000 m, and 2500 and 3500 m, were used for testing, respectively. Classification is only performed when there is detection in a frame. The observations are summarized in Table 6 and another two tables in Appendix D. In each table, the left side includes a confusion matrix and the last column contains the classification accuracy. From Table 6 (PCE full), one can see that accuracy is not great but decent. For PCE 50 and PCE 25 cases, the performance drops quite significantly, as can be seen from the tables in Appendix D.

If one compares the optical results in Section 4 and results here, one can observe that the optical results are better than the MWIR in daytime.

### 5.3. Summary (MWIR Daytime)

It is important to emphasize that we are tackling a challenging problem in target tracking and classification in long range and low quality videos. The SENSIAC videos are difficult to track and classify even in the uncompressed case. Here, we condense the results in Table 5 and Table 6 and those additional tables in Appendix C and Appendix D in Table 7. For daytime videos using the MWIR imager, the tracking performance is only good for PCE full and up to 2000 m. For classification, the results are poor in general even for PCE full case. A simple comparison with the optical results in Table 4 concludes that MWIR is not recommended for daytime tracking and classification.

## 6. MWIR Nighttime Videos

This section focuses on MWIR nighttime videos.

### 6.1. Tracking

**Figure 11:**
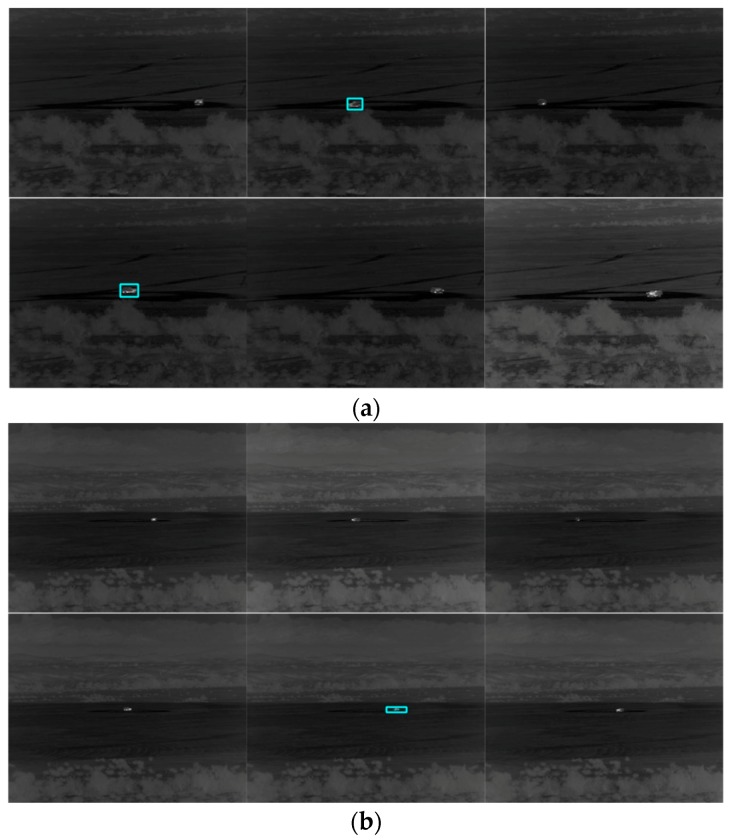

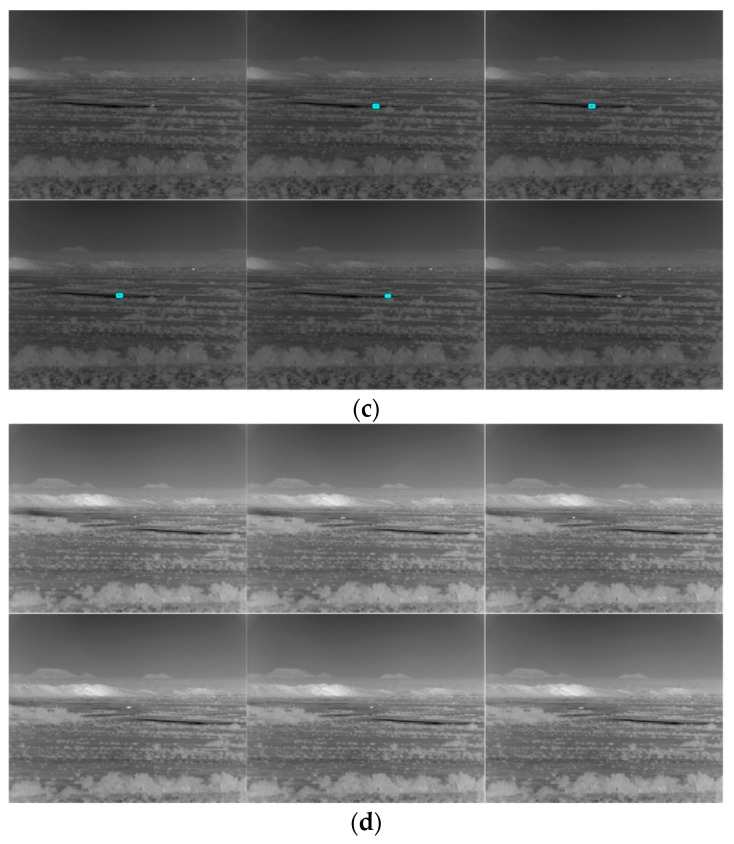
Tracking results for frames 1, 60, 119, 178, 237, and 296 for the PCE full (MWIR nighttime) case. The vehicle is SUV. (**a**) 1000 m; (**b**) 2000 m; (**c**) 2500 m; and (**d**) 3500 m.

We built two models using videos from 1500 m and 3000 m. For the 1500 m model, videos from 1000 m and 2000 m were used for testing. For the 3000 m model, we used videos from 2500 m and 3500 m for testing. Table 8 and two additional tables in Appendix E show the tracking results for PCE full, PCE 50, and PCE 25, respectively. For PCE full case, the results in Table 8 show that the tracking results are quite good. For the PCE 50 and PCE 25 cases, the results in those tables in Appendix E drop quite significantly. The trend is that when the image compression ratio increases, the performance drops accordingly. In the long range cases (Table 8 and the tables in Appendix E), one can observe some numbers of 0% detection and no detection (ND) cases. This is understandable because MWIR imagers rely of radiation from the target and if the target is far, the signal to noise ratio (SNR) is very low for long ranges. Hence, the target signals will be very weak in long ranges. This can be confirmed in the snapshots shown in Figure 11 and two additional figures in Appendix E where we can see that some targets do not have bounding boxes around them in the high compression cases.

### 6.2. Classification

Classification is only done when there is detection in a frame. Two classifiers were built: one for 1500 m and one for 3000 m. For PCE full case (Table 9), the classification performance is good for ranges up to 2000 m. For longer ranges, the performance drops. For PCE 50 and PCE 25 results shown in those tables in Appendix F, the longer ranges (≥2500 m) are very poor. As mentioned earlier, MWIR imager relies on signals from the targets and long ranges make the signal very weak. Consequently, the overall tracking and classification results are not good.

### 6.3. Summary (MWIR Nighttime)

Table 10 summarizes the averaged classification accuracy of the various cases presented earlier. It can be seen the if one is interested in highly accurate classification, then the range has to be less than 2000 m and we need to adopt PCE full mode. Moreover, when we compare the results of MWIR daytime and nighttime results, we will observe that the nighttime results are better. Hence, MWIR should be recommended for nighttime tracking and classification.

## 7. Conclusions

In this paper, we present a deep learning based approach to target tracking and classification directly using PCE measurements. No time consuming reconstruction step is needed and hence real-time target tracking and classification is possible for practical applications. The proposed approach is based on a combination of two deep learning schemes: YOLO for tracking and ResNet for classification. Comparing with state-of-the-art methods, which either assume the objects are cropped and centered or are only applicable to action inference rather than object classification, our approach is suitable for target tracking and classification applications where limited training data are available. Extensive experiments using 378 optical and MWIR (daytime and nighttime) videos with different ranges, illumination, and environmental conditions in the SENSIAC database clearly demonstrated the performance. Moreover, it was observed that optical is more suitable for daytime operations and MWIR is more appropriate for nighttime operations.

It should be emphasized that the SENSIAC database is very challenging for target tracking and classification, even when using the original measurements. There are some videos collected beyond 3500 m that we have not even touched in our paper. More research is needed for the research community to address such challenging scenarios.

## Figures and Tables

**Table 1:** Comparison in Data Compression Ratio and Power Saving Ratio between Three Sensing Models. Here, 30 frames are condensed to 1 coded frame.

Savings	PCE Full/CA Full	PCE 50%/CA 50%	PCE 25%/CA 25%
Data Saving Ratio	30:1	60:1	120:1
Power Saving Ratio	1:1	15:1	30:1

**Table 2:** Tracking metrics for PCE full (optical videos).

**1000 m**					**2500 m**				
**Vehicles**	**EinGT**	**CLE**	**DP@20 Pixels**	**% Detections**	**Vehicles**	**EinGT**	**CLE**	**DP@20 Pixels**	**% Detections**
BMP2	1	39.96	0%	100	BMP2	1	14.27	100%	1%
BRDM2	1	23.54	14%	98	BRDM2	1	7.96	100%	44%
BTR70	1	31.06	0%	100	BTR70	1	11.32	100%	40%
SUV	1	27.25	0%	100	SUV	1	9.58	100%	46%
T72	1	63.86	0%	100	T72	1	22.46	2%	44%
Truck	1	26.36	1%	99	Truck	1	9.92	100%	8%
ZSU23-4	1	37.29	0%	99	ZSU23-4	1	13.07	100%	82%
**1500 m**					**3000 m**				
**Vehicles**	**EinGT**	**CLE**	**DP@20 Pixels**	**% Detections**	**Vehicles**	**EinGT**	**CLE**	**DP@20 Pixels**	**% Detections**
BMP2	1	28.08	0%	100%	BMP2	1	8.39	100%	77%
BRDM2	1	15.79	100%	100%	BRDM2	1	4.81	100%	100%
BTR70	1	21.94	11%	100%	BTR70	1	7.04	100%	100%
SUV	1	20.16	47%	100%	SUV	1	6.05	100%	73%
T72	1	46.96	0%	100%	T72	1	15.25	100%	86%
Truck	1	20.59	36%	100%	Truck	1	6.3	100%	100%
ZSU23-4	1	26.93	0%	100%	ZSU23-4	1	7.98	100%	100%
**2000 m**					**3500 m**				
**Vehicles**	**EinGT**	**CLE**	**DP@20 Pixels**	**% Detections**	**Vehicles**	**EinGT**	**CLE**	**DP@20 Pixels**	**% Detections**
BMP2	1	18.86	85%	100%	BMP2	0	0	0%	0%
BRDM2	1	9.59	100%	100%	BRDM2	0.41	7.96	100%	23%
BTR70	1	15.23	100%	100%	BTR70	1	4.74	100%	20%
SUV	1	12.69	100%	100%	SUV	0.58	2.51	100%	11%
T72	1	31.41	0%	100%	T72	1	8.76	100%	5%
Truck	1	13.03	100%	100%	Truck	0.87	3.98	100%	14%
ZSU23-4	1	19.15	71%	100%	ZSU23-4	0.96	4.4	100%	30%

**Table 3:** Classification results for PCE full (optical) case. Left shows the confusion matrix and the last column shows the classification accuracy.

**1000 m**									**2500 m**								
**Vehicles**	**BMP2**	**BRDM2**	**BTR70**	**SUV**	**T72**	**Truck**	**ZSU23-4**	**Accuracy**	**Vehicles**	**BMP2**	**BRDM2**	**BTR70**	**SUV**	**T72**	**Truck**	**ZSU23-4**	**Accuracy**
BMP2	366	1	0	2	1	0	4	98%	BMP2	0	0	0	0	3	0	0	0%
BRDM2	0	210	0	4	1	152	0	57%	BRDM2	0	56	0	0	0	107	1	34%
BTR70	0	0	373	0	0	1	0	100%	BTR70	24	0	86	1	6	0	32	58%
SUV	0	0	0	189	0	185	0	51%	SUV	0	0	23	78	48	18	4	46%
T72	0	15	0	38	310	11	0	83%	T72	0	0	0	0	160	3	0	98%
Truck	10	6	0	38	0	315	0	85%	Truck	1	0	4	0	19	5	0	17%
ZSU23-4	0	0	0	10	0	0	359	97%	ZSU23-4	0	0	0	0	100	0	205	67%
**1500 m**									**3000 m**								
**Vehicles**	**BMP2**	**BRDM2**	**BTR70**	**SUV**	**T72**	**Truck**	**ZSU23-4**	**Accuracy**	**Vehicles**	**BMP2**	**BRDM2**	**BTR70**	**SUV**	**T72**	**Truck**	**ZSU23-4**	**Accuracy**
BMP2	363	0	0	11	0	0	0	97%	BMP2	0	0	139	1	149	0	0	0%
BRDM2	0	234	0	76	0	64	0	63%	BRDM2	14	113	13	17	2	209	6	30%
BTR70	0	0	374	0	0	0	0	100%	BTR70	47	0	260	0	58	0	9	70%
SUV	0	0	0	201	0	173	0	54%	SUV	1	0	264	1	8	0	0	0%
T72	0	4	1	0	369	0	0	99%	T72	0	0	84	7	211	19	0	66%
Truck	3	10	0	0	0	361	0	97%	Truck	38	0	47	0	153	131	5	35%
ZSU23-4	0	0	0	0	0	0	374	100%	ZSU23-4	5	0	27	8	268	0	66	18%
**2000 m**									**3500 m**								
**Vehicles**	**BMP2**	**BRDM2**	**BTR70**	**SUV**	**T72**	**Truck**	**ZSU23-4**	**Accuracy**	**Vehicles**	**BMP2**	**BRDM2**	**BTR70**	**SUV**	**T72**	**Truck**	**ZSU23-4**	**Accuracy**
BMP2	355	0	0	17	0	2	0	95%	BMP2	0	0	0	0	0	0	0	0%
BRDM2	0	40	9	170	0	155	0	11%	BRDM2	20	4	28	1	6	28	0	5%
BTR70	2	0	355	15	0	2	0	95%	BTR70	0	0	70	0	6	0	0	92%
SUV	1	0	2	97	0	258	8	27%	SUV	4	0	27	1	2	6	0	3%
T72	4	26	1	194	89	62	1	24%	T72	0	0	2	0	9	9	0	45%
Truck	17	56	4	2	1	285	1	78%	Truck	2	0	18	0	2	31	0	58%
ZSU23-4	0	1	41	14	2	28	287	77%	ZSU23-4	0	0	5	0	102	4	0	0%

**Table 4:** Averaged tracking and classification performances for the various optical video cases. 1500 m and 3000 m videos were used for training.

PCE Full			PCE 50			PCE 25		
Range	Average % of Frames with Detections	Average Accuracy	Range	Average % of Frames with Detections	Average Accuracy	Range	Average % of Frames with Detections	Average Accuracy
1000	99%	82%	1000	79%	52%	1000	59%	39%
1500	100%	87%	1500	99%	53%	1500	59%	39%
2000	99%	58%	2000	71%	27%	2000	27%	29%
2500	38%	46%	2500	0%	0%	2500	1%	0%
3000	91%	31%	3000	2%	16%	3000	6%	16%
3500	15%	29%	3500	0%	0%	3500	2%	18%

**Table 5:** Tracking metrics for PCE full (MWIR daytime) case. 1500 m and 3000 m were used for training.

**1000 m**					**2500 m**				
**Vehicles**	**EinGT**	**CLE**	**DP@20 Pixels**	**% Detections**	**Vehicles**	**EinGT**	**CLE**	**DP@20 Pixels**	**% Detections**
BMP2	1.00	29.46	0%	52%	BMP2	1.00	10.01	100%	82%
BRDM2	1.00	24.86	4%	94%	BRDM2	1.00	9.73	100%	75%
BTR70	1.00	24.65	13%	69%	BTR70	0.80	35.19	80%	35%
SUV	1.00	18.54	69%	81%	SUV	0.99	10.47	99%	22%
T72	1.00	34.30	0%	53%	T72	0.99	13.04	99%	60%
Truck	1.00	23.61	5%	58%	Truck	0.99	10.38	99%	31%
ZSU23-4	1.00	29.41	0%	42%	ZSU23-4	1.00	10.51	100%	65%
**1500 m**					**3000 m**				
**Vehicles**	**EinGT**	**CLE**	**DP@20 Pixels**	**% Detections**	**Vehicles**	**EinGT**	**CLE**	**DP@20 Pixels**	**% Detections**
BMP2	1.00	22.32	10%	99%	BMP2	1.00	7.20	100%	100%
BRDM2	1.00	18.87	82%	99%	BRDM2	1.00	6.35	100%	100%
BTR70	1.00	17.94	95%	99%	BTR70	1.00	5.93	100%	100%
SUV	1.00	13.89	100%	90%	SUV	1.00	4.60	100%	100%
T72	1.00	24.86	0%	97%	T72	1.00	7.88	100%	100%
Truck	1.00	17.42	90%	95%	Truck	1.00	5.48	100%	100%
ZSU23-4	1.00	20.77	32%	99%	ZSU23-4	1.00	6.63	100%	100%
**2000 m**					**3500 m**				
**Vehicles**	**EinGT**	**CLE**	**DP@20 Pixels**	**% Detections**	**Vehicles**	**EinGT**	**CLE**	**DP@20 Pixels**	**% Detections**
BMP2	1.00	15.32	100%	77%	BMP2	1.00	4.41	100%	33%
BRDM2	1.00	12.63	100%	64%	BRDM2	0.17	2.25	100%	52%
BTR70	1.00	11.12	100%	86%	BTR70	0.97	4.51	99%	31%
SUV	1.00	9.53	100%	31%	SUV	0.97	1.83	100%	33%
T72	1.00	16.88	99%	64%	T72	0.86	4.91	100%	70%
Truck	1.00	11.87	100%	30%	Truck	1.00	3.48	100%	11%
ZSU23-4	1.00	13.45	100%	93%	ZSU23-4	1.00	3.36	100%	36%

**Table 6:** Classification results for PCE Full (MWIR daytime) case. Left shows the confusion matrix and the last column shows the classification accuracy.

**1000 m**									**2500 m**								
**Vehicles**	**BMP2**	**BRDM2**	**BTR70**	**SUV**	**T72**	**Truck**	**ZSU23-4**	**Accuracy**	**Vehicles**	**BMP2**	**BRDM2**	**BTR70**	**SUV**	**T72**	**Truck**	**ZSU23-4**	**Accuracy**
BMP2	123	15	27	0	15	2	3	66%	BMP2	283	2	6	1	2	0	0	96%
BRDM2	93	224	7	4	3	8	0	66%	BRDM2	1	194	0	8	50	3	12	72%
BTR70	72	6	158	4	0	6	0	64%	BTR70	3	36	56	16	5	5	4	45%
SUV	29	1	0	244	3	14	0	84%	SUV	0	19	5	13	19	11	12	16%
T72	106	9	0	1	72	3	0	38%	T72	8	70	19	11	68	31	8	32%
Truck	54	0	0	1	0	154	0	74%	Truck	4	8	0	34	32	8	24	7%
ZSU23-4	36	40	9	5	0	28	33	22%	ZSU23-4	0	4	1	5	0	1	221	95%
**1500 m**									**3000 m**								
**Vehicles**	**BMP2**	**BRDM2**	**BTR70**	**SUV**	**T72**	**Truck**	**ZSU23-4**	**Accuracy**	**Vehicles**	**BMP2**	**BRDM2**	**BTR70**	**SUV**	**T72**	**Truck**	**ZSU23-4**	**Accuracy**
BMP2	82	2	6	7	7	236	16	23%	BMP2	344	1	0	1	1	5	7	96%
BRDM2	5	330	2	0	0	20	0	92%	BRDM2	1	268	0	57	23	4	6	75%
BTR70	46	0	215	1	7	85	1	61%	BTR70	0	110	104	39	38	7	61	29%
SUV	1	4	0	247	1	68	2	76%	SUV	18	24	4	219	42	24	28	61%
T72	112	35	0	6	179	18	0	51%	T72	11	9	0	67	216	49	7	60%
Truck	3	14	0	12	9	292	10	86%	Truck	20	59	10	116	60	75	19	21%
ZSU23-4	5	76	0	0	15	130	130	37%	ZSU23-4	1	7	9	13	2	5	322	90%
**2000 m**									**3500 m**								
**Vehicles**	**BMP2**	**BRDM2**	**BTR70**	**SUV**	**T72**	**Truck**	**ZSU23-4**	**Accuracy**	**Vehicles**	**BMP2**	**BRDM2**	**BTR70**	**SUV**	**T72**	**Truck**	**ZSU23-4**	**Accuracy**
BMP2	87	0	3	10	20	149	9	31%	BMP2	101	0	5	3	6	3	1	85%
BRDM2	39	81	0	0	22	76	13	35%	BRDM2	20	76	1	51	17	3	17	41%
BTR70	5	65	117	0	25	0	97	38%	BTR70	2	16	1	27	10	2	53	1%
SUV	20	14	0	8	66	1	2	7%	SUV	2	7	0	64	27	5	12	55%
T72	4	19	0	0	177	13	17	77%	T72	24	27	5	5	167	17	7	66%
Truck	0	0	0	0	30	78	1	72%	Truck	6	5	0	21	1	5	1	13%
ZSU23-4	0	0	0	0	6	0	329	98%	ZSU23-4	1	19	4	7	13	3	84	64%

**Table 7:** Average detection and classification performance of different MWIR daytime cases. 1500 m and 3000 m were used for training.

PCE Full			PCE 50			PCE 25		
Range	Average % of Frames with Detections	Average Accuracy	Range	Average % of Frames with Detections	Average Accuracy	Range	Average % of Frames with Detections	Average Accuracy
1000	64%	59%	1000	18%	49%	1000	28%	42%
1500	97%	61%	1500	60%	43%	1500	61%	42%
2000	64%	51%	2000	6%	18%	2000	4%	26%
2500	53%	52%	2500	6%	19%	2500	29%	16%
3000	100%	62%	3000	52%	31%	3000	12%	26%
3500	38%	46%	3500	11%	14%	3500	10%	4%

**Table 8:** Tracking metrics for PCE full (MWIR nighttime).

**1000 m**					**2500 m**					
**Vehicles**	**EinGT**	**CLE**	**DP@20 Pixels**	**% Detections**	**Vehicles**	**EinGT**	**CLE**	**DP@20 Pixels**	**% Detections**	
BMP2	1.00	29.10	1%	52%	BMP2	0.99	0.99	12.09	99%	96%
BRDM2	1.00	25.71	8%	77%	BRDM2	1.00	1.00	9.41	100%	100%
BTR70	1.00	17.80	74%	90%	BTR70	1.00	1.00	5.43	100%	100%
SUV	1.00	14.31	100%	99%	SUV	1.00	1.00	5.00	100%	100%
T72	1.00	34.43	0%	65%	T72	1.00	1.00	10.77	100%	100%
Truck	1.00	26.19	2%	79%	Truck	1.00	1.00	9.37	100%	90%
ZSU23-4	1.00	27.96	0%	80%	ZSU23-4	1.00	1.00	9.93	100%	90%
**1500 m**					**3000 m**					
**Vehicles**	**EinGT**	**CLE**	**DP@20 Pixels**	**% Detections**	**Vehicles**	**EinGT**	**CLE**	**DP@20 Pixels**	**% Detections**	
BMP2	1.00	19.92	50%	100%	BMP2	1.00	6.81	100%	100%	
BRDM2	1.00	19.63	63%	94%	BRDM2	1.00	6.62	100%	99%	
BTR70	1.00	11.86	100%	97%	BTR70	1.00	3.86	100%	100%	
SUV	1.00	10.52	100%	97%	SUV	1.00	4.10	100%	91%	
T72	1.00	23.91	1%	100%	T72	1.00	7.43	100%	100%	
Truck	1.00	19.32	71%	97%	Truck	1.00	7.33	100%	99%	
ZSU23-4	1.00	19.57	62%	87%	ZSU23-4	1.00	6.91	100%	100%	
**2000 m**					**3500 m**					
**Vehicles**	**EinGT**	**CLE**	**DP@20 Pixels**	**% Detections**	**Vehicles**	**EinGT**	**CLE**	**DP@20 Pixels**	**% Detections**	
BMP2	1.00	13.85	100%	66%	BMP2	1.00	3.19	100%	11%	
BRDM2	1.00	14.00	100%	65%	BRDM2	0.98	3.41	99%	54%	
BTR70	1.00	7.91	100%	55%	BTR70	0.94	2.29	100%	60%	
SUV	1.00	6.47	100%	84%	SUV	0.96	2.19	100%	51%	
T72	1.00	16.70	98%	93%	T72	0.93	4.91	100%	86%	
Truck	1.00	12.38	100%	25%	Truck	0.74	12.96	93%	64%	
ZSU23-4	1.00	13.42	100%	78%	ZSU23-4	0.99	4.40	100%	96%	

**Table 9:** Classification results for PCE full case (MWIR nighttime). Left shows the confusion matrix and the last column shows the classification accuracy.

**1000 m**									**2500 m**								
**Vehicles**	**BMP2**	**BRDM2**	**BTR70**	**SUV**	**T72**	**Truck**	**ZSU23-4**	**Accuracy**	**Vehicles**	**BMP2**	**BRDM2**	**BTR70**	**SUV**	**T72**	**Truck**	**ZSU23-4**	**Accuracy**
BMP2	184	0	0	0	1	0	0	99%	BMP2	162	35	0	0	81	6	60	47%
BRDM2	0	274	0	0	0	3	0	99%	BRDM2	5	288	0	1	25	39	1	80%
BTR70	24	21	234	0	6	39	0	72%	BTR70	0	40	130	5	28	139	16	36%
SUV	0	0	0	347	0	9	0	97%	SUV	2	7	6	263	27	50	4	73%
T72	0	0	1	1	224	5	2	96%	T72	12	1	0	4	305	31	5	85%
Truck	3	0	0	1	7	271	0	96%	Truck	46	10	9	7	28	221	3	68%
ZSU23-4	0	1	0	2	0	1	283	99%	ZSU23-4	2	21	4	2	11	9	274	85%
**1500 m**									**3000 m**								
**Vehicles**	**BMP2**	**BRDM2**	**BTR70**	**SUV**	**T72**	**Truck**	**ZSU23-4**	**Accuracy**		**BMP2**	**BRDM2**	**BTR70**	**SUV**	**T72**	**Truck**	**ZSU23-4**	**Accuracy**
BMP2	341	10	2	0	3	3	0	95%	BMP2	202	54	1	0	93	1	8	56%
BRDM2	0	329	0	0	10	0	0	97%	BRDM2	9	271	2	7	26	33	8	76%
BTR70	0	1	347	0	0	0	0	100%	BTR70	0	31	233	9	7	1	78	65%
SUV	0	1	0	277	5	59	5	80%	SUV	3	0	0	316	6	0	0	97%
T72	3	0	0	0	352	3	0	98%	T72	40	17	8	4	249	34	7	69%
Truck	3	1	4	1	26	312	1	90%	Truck	2	12	0	14	25	302	2	85%
ZSU23-4	0	3	1	1	3	2	301	97%	ZSU23-4	7	10	0	1	7	2	332	92%
**2000 m**									**3500 m**								
**Vehicles**	**BMP2**	**BRDM2**	**BTR70**	**SUV**	**T72**	**Truck**	**ZSU23-4**	**Accuracy**	**Vehicles**	**BMP2**	**BRDM2**	**BTR70**	**SUV**	**T72**	**Truck**	**ZSU23-4**	**Accuracy**
BMP2	13	10	0	0	205	8	1	5%	BMP2	17	8	0	0	4	9	2	43%
BRDM2	0	207	0	0	23	4	0	88%	BRDM2	1	146	2	1	24	15	5	75%
BTR70	0	12	176	0	10	0	0	89%	BTR70	2	12	79	10	1	37	76	36%
SUV	1	0	6	208	13	72	0	69%	SUV	19	0	1	119	19	4	20	65%
T72	28	0	0	0	296	9	1	89%	T72	35	57	5	25	142	20	26	46%
Truck	0	2	1	0	15	69	1	78%	Truck	7	27	1	44	38	114	0	49%
ZSU23-4	3	5	7	4	65	31	165	59%	ZSU23-4	2	5	5	3	34	1	296	86%

**Table 10:** Averaged classification results at PCE full, PCE 50, and PCE 25 for the MWIR nighttime videos. 1500 m and 3000 m were used for training.

PCE Full			PCE 50			PCE 25		
Range	Average % of Frames with Detections	Average Accuracy	Range	Average % of Frames with Detections	Average Accuracy	Range	Average % of Frames with Detections	Average Accuracy
1000	77%	94%	1000	77%	67%	1000	96%	64%
1500	96%	94%	1500	93%	67%	1500	100%	64%
2000	66%	68%	2000	54%	41%	2000	63%	48%
2500	96%	68%	2500	0%	0%	2500	0%	0%
3000	98%	77%	3000	0%	0%	3000	0%	0%
3500	64%	49%	3500	0%	0%	3500	0%	0%

**Figure A1:**
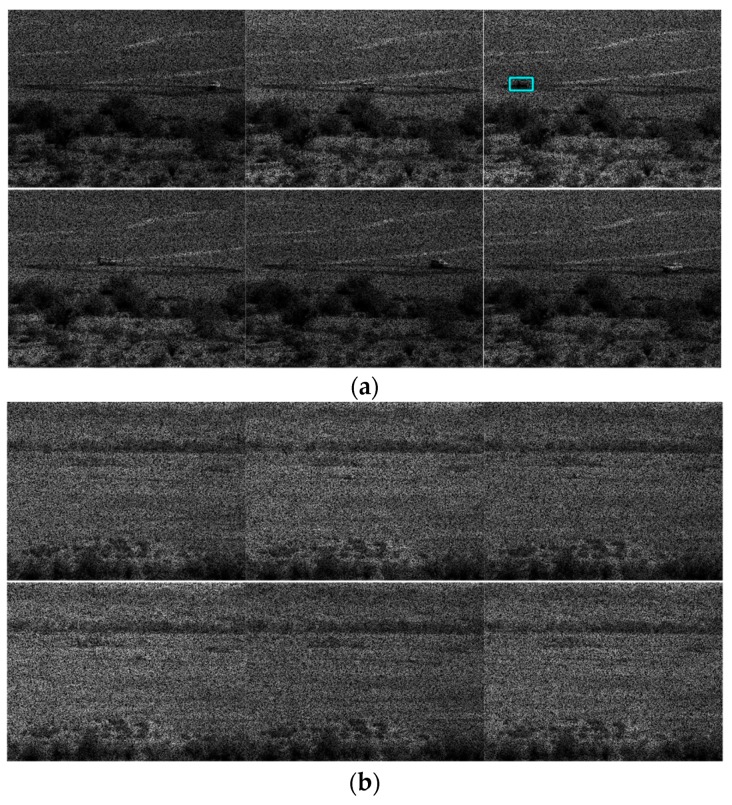

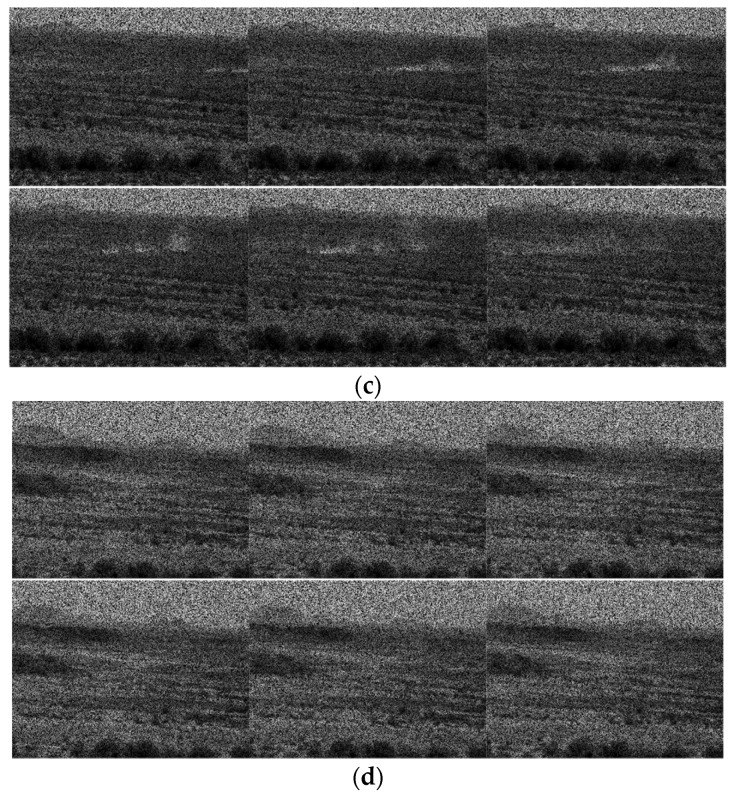
Tracking results for frames 1, 63, 126, 189, 252, and 315 for the PCE 50 (optical video) case. The vehicle is SUV. Most of the captured frames do not have detection. Dusts can be seen in some frames and have serious impacts on the tracking and classification performance. (**a**) 1000 m; (**b**) 2000 m; (**c**) 2500 m; and (**d**) 3500 m.

**Figure A2:**
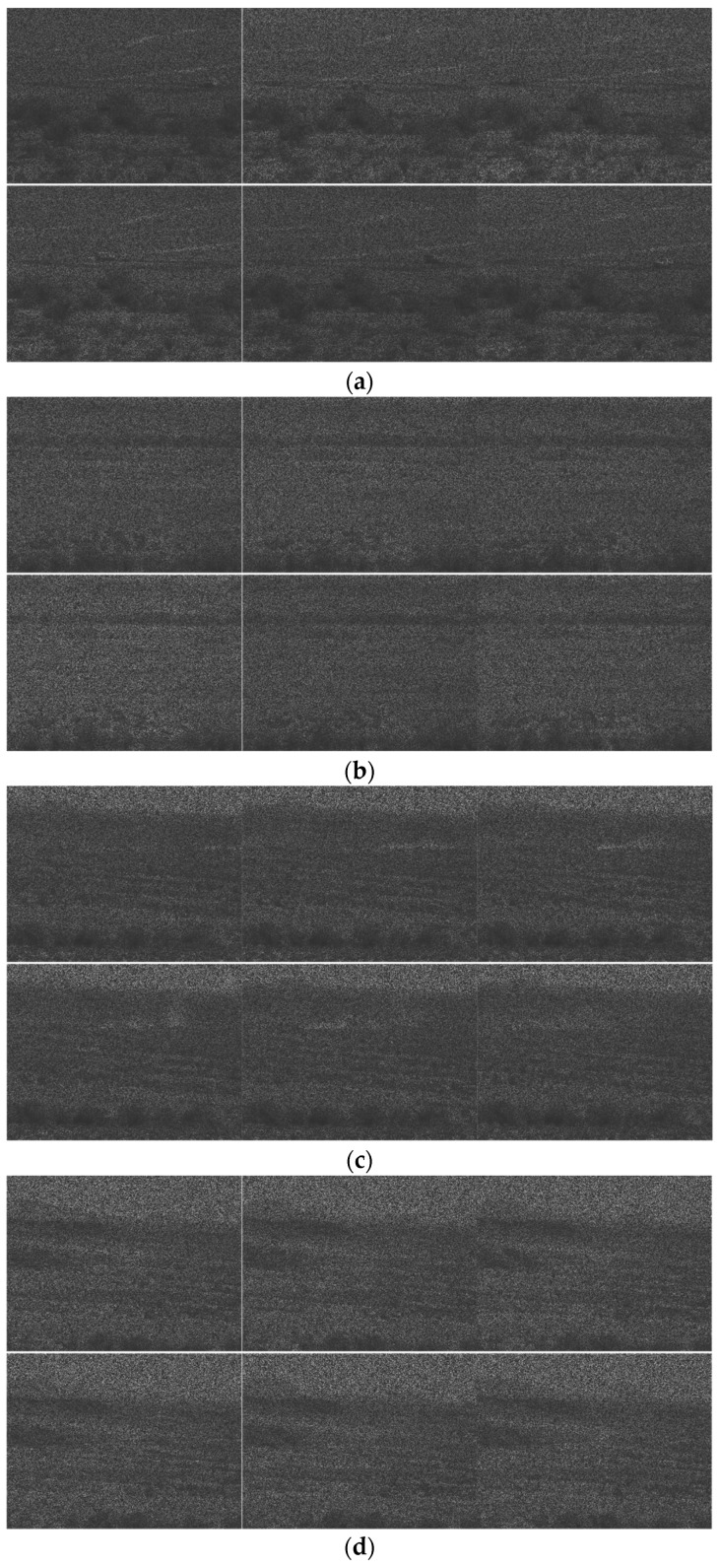
Tracking results for frames 1, 63, 126, 189, 252, and 315 for the PCE 25 (optical video) case. The vehicle is SUV. No detections are observed in the sampled frames. (**a**) 1000 m; (**b**) 2000 m; (**c**) 2500 m; and (**d**) 3500 m.

**Figure A3:**
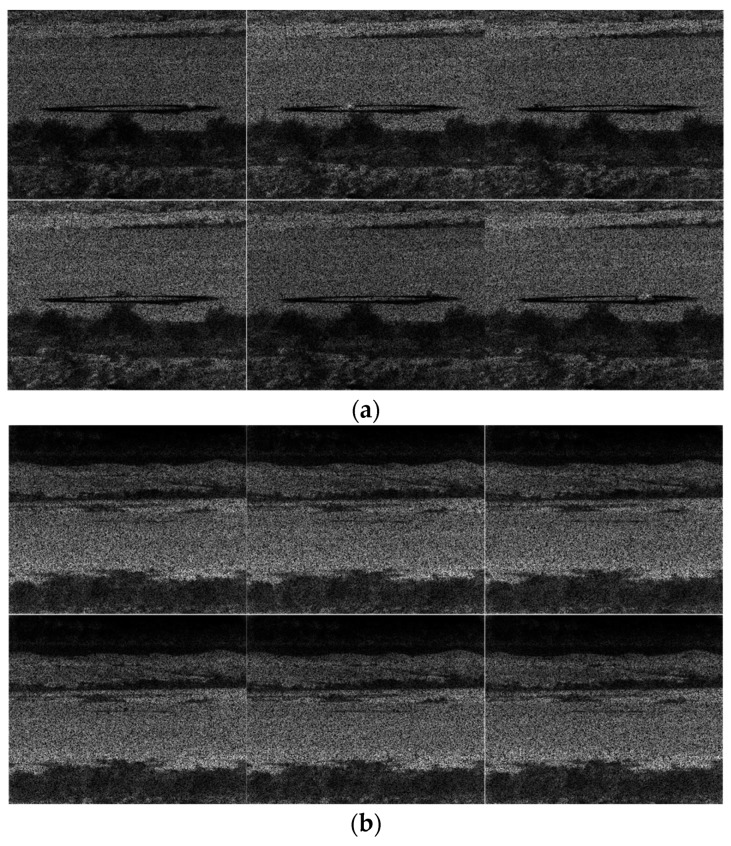

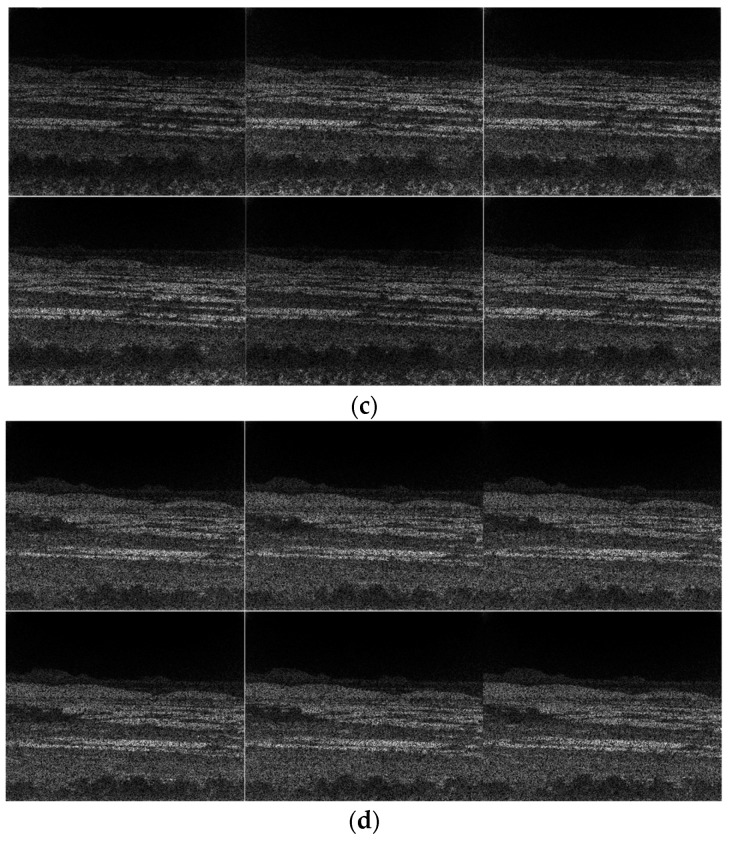
Tracking results for frames 1, 60, 119, 178, 237, and 296 for the PCE 50 (MWIR daytime) case. The vehicle is SUV. No detection is observed in the sampled frames. (**a**) 1000 m; (**b**) 2000 m; (**c**) 2500 m; and (**d**) 3500 m.

**Figure A4:**
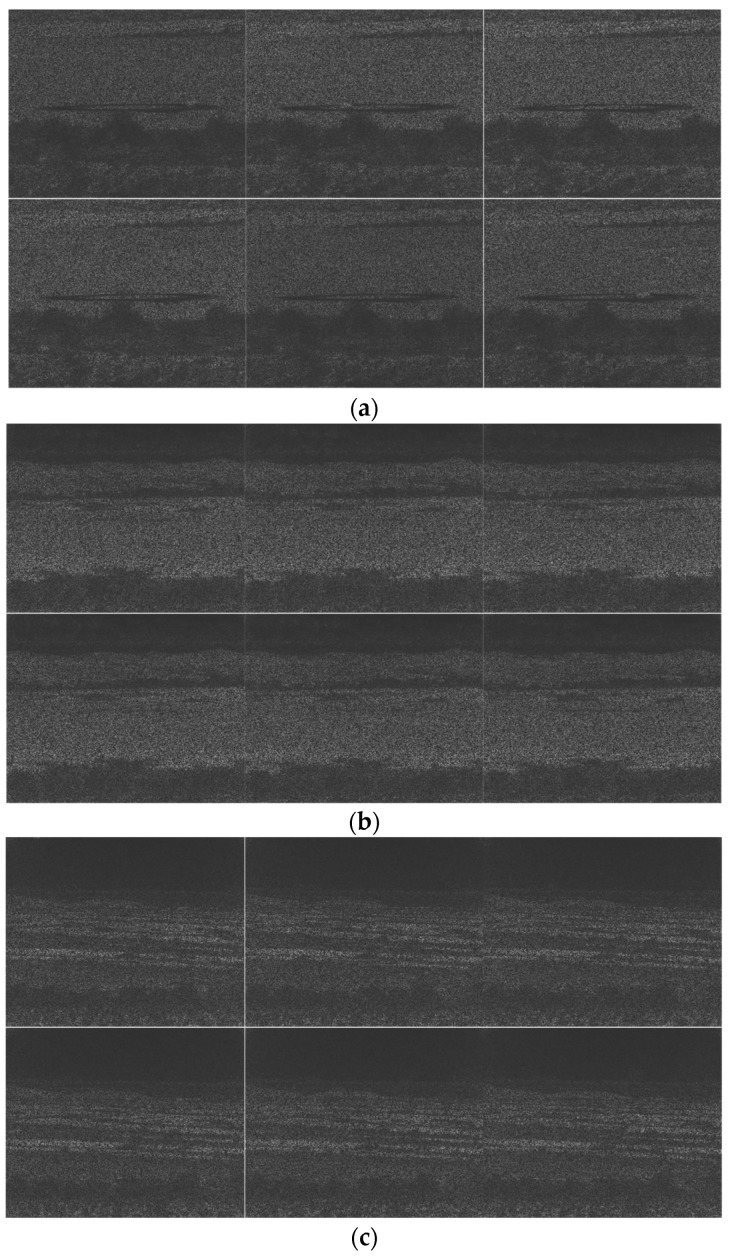

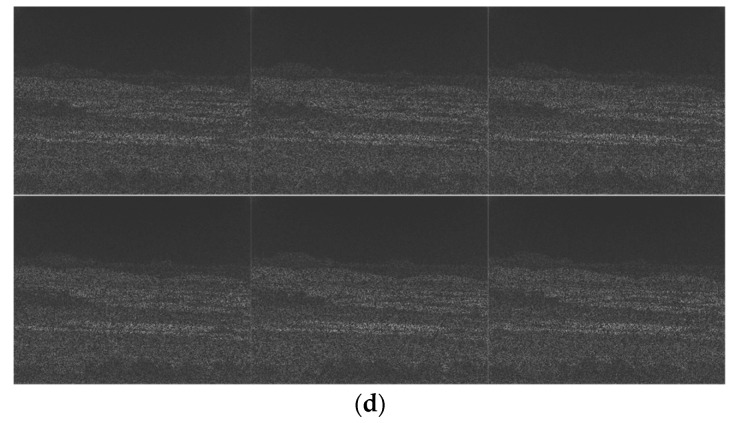
Tracking results for frames 1, 60, 119, 178, 237, and 296 for the PCE 25 (MWIR daytime) case. The vehicle is SUV. No detections can be seen in the sampled frames. (**a**) 1000 m; (**b**) 2000 m; (**c**) 2500 m; and (**d**) 3500 m.

**Figure A5:**
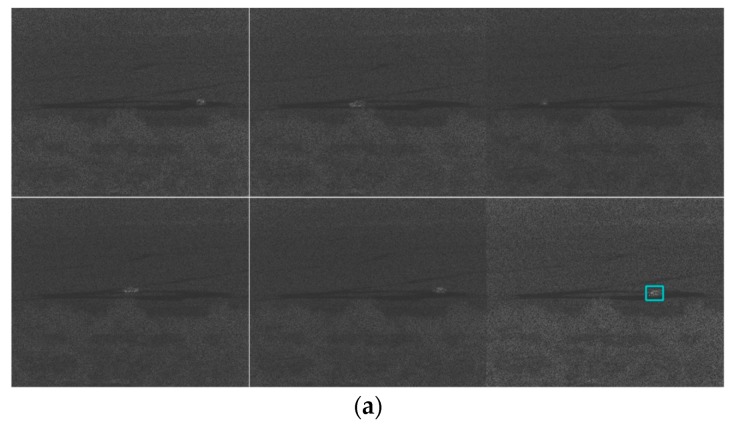

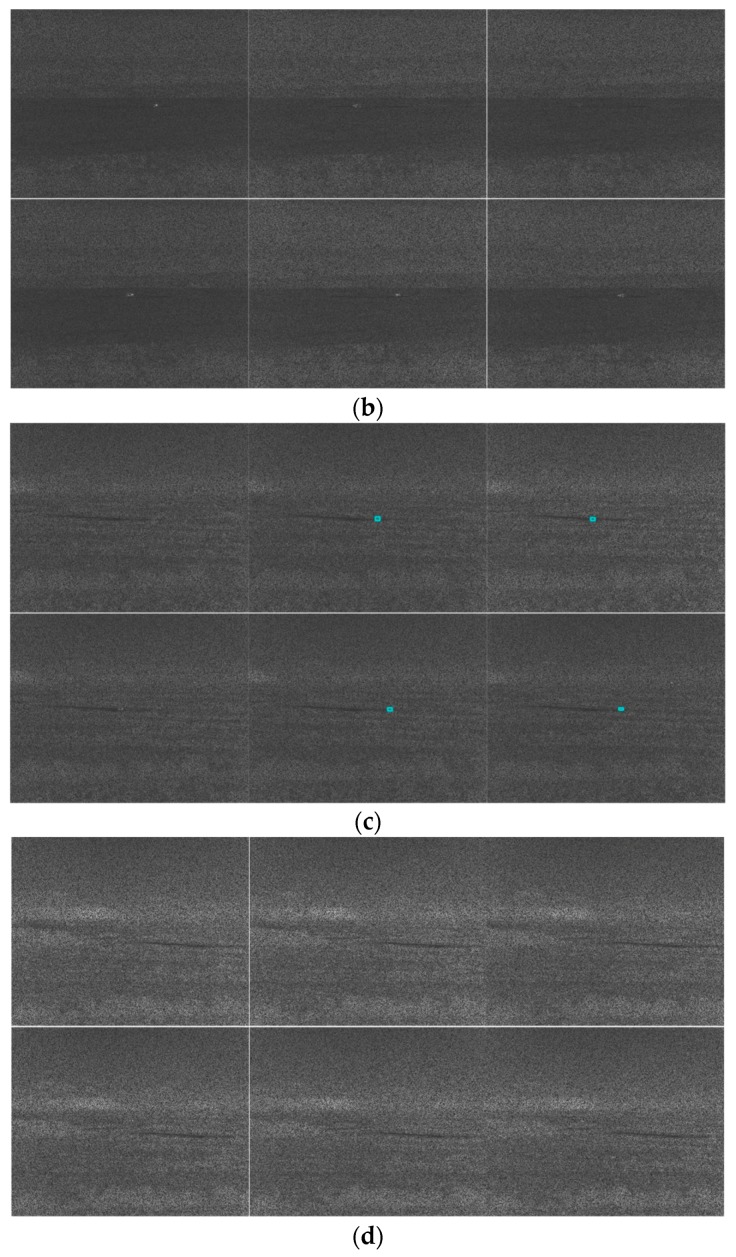
Tracking results for frames 1, 60, 119, 178, 237, and 296 for the PCE 50 (MWIR nighttime) case. The vehicle is SUV. (**a**) 1000 m; (**b**) 2000 m; (**c**) 2500 m; and (**d**) 3500 m.

**Figure A6:**
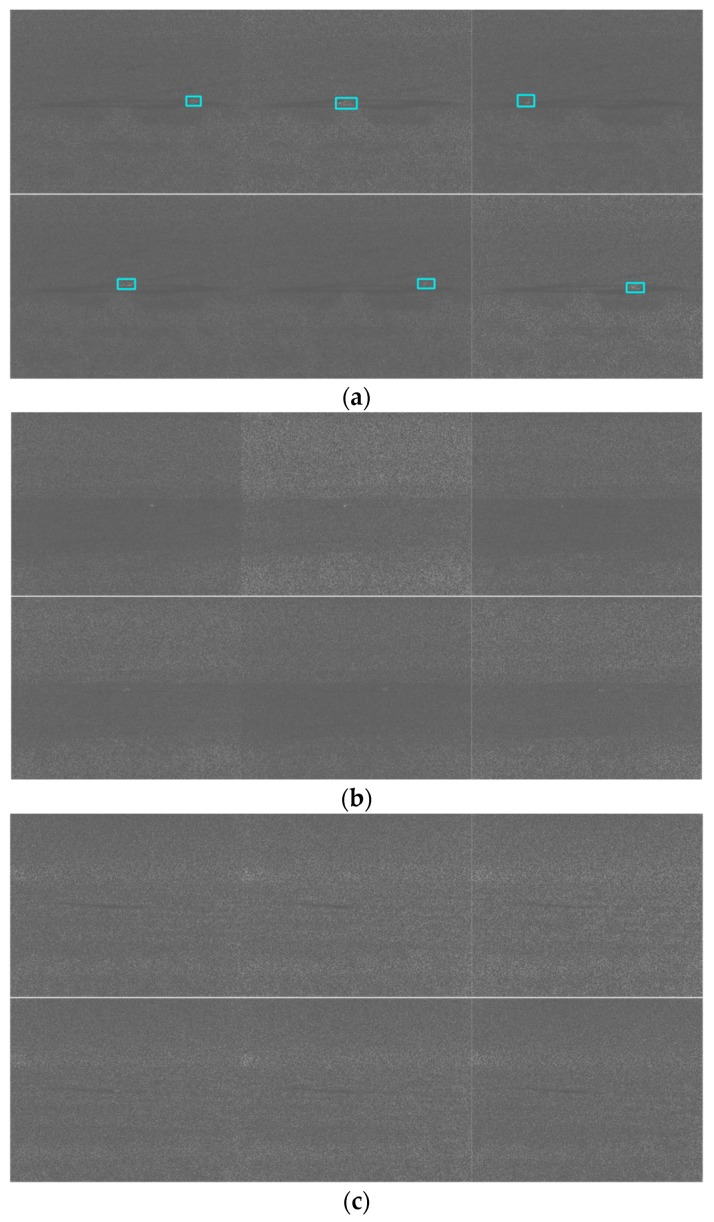

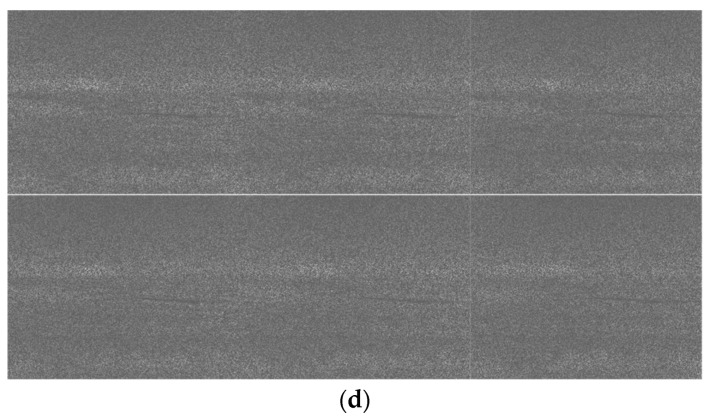
Tracking results for frames 1, 60, 119, 178, 237, and 296 for the PCE 25 (MWIR nighttime) case. The vehicle is SUV. (**a**) 1000 m; (**b**) 2000 m; (**c**) 2500 m; and (**d**) 3500 m.

## Appendix A. Tracking Results for Optical Case: PCE 50 and PCE 25

**Table A1:** Tracking metrics for PCE 50 (optical video). ND means “no detection”.

**1000 m**					**2500 m**				
**Vehicles**	**EinGT**	**CLE**	**DP@20 Pixels**	**% Detections**	**Vehicles**	**EinGT**	**CLE**	**DP@20 Pixels**	**% Detections**
BMP2	1.00	39.25	0%	75%	BMP2	0.00	ND	0%	0%
BRDM2	1.00	19.36	66%	74%	BRDM2	0.00	ND	0%	0%
BTR70	1.00	29.46	0%	83%	BTR70	0.00	ND	0%	0%
SUV	1.00	24.95	6%	78%	SUV	0.00	ND	0%	0%
T72	1.00	61.78	0%	90%	T72	0.00	ND	0%	0%
Truck	1.00	24.95	11%	79%	Truck	0.00	ND	0%	0%
ZSU23-4	1.00	33.57	0%	75%	ZSU23-4	0.00	ND	0%	0%
**1500 m**					**3000 m**				
**Vehicles**	**EinGT**	**CLE**	**DP@20 Pixels**	**% Detections**	**Vehicles**	**EinGT**	**CLE**	**DP@20 Pixels**	**% Detections**
BMP2	1.00	27.77	0%	100%	BMP2	0.00	ND	0%	0%
BRDM2	1.00	15.00	97%	96%	BRDM2	1.00	5.93	100%	2%
BTR70	1.00	21.95	21%	100%	BTR70	1.00	7.27	100%	6%
SUV	1.00	18.83	71%	100%	SUV	0.00	ND	0%	0%
T72	1.00	46.71	0%	100%	T72	1.00	14.24	100%	1%
Truck	1.00	19.82	50%	100%	Truck	1.00	5.03	100%	1%
ZSU23-4	1.00	24.89	1%	100%	ZSU23-4	1.00	7.84	100%	4%
**2000 m**					**3500 m**				
**Vehicles**	**EinGT**	**CLE**	**DP@20 Pixels**	**% Detections**	**Vehicles**	**EinGT**	**CLE**	**DP@20 Pixels**	**% Detections**
BMP2	1.00	19.85	54%	95%	BMP2	0.00	ND	0%	0%
BRDM2	1.00	10.92	100%	5%	BRDM2	0.00	ND	0%	0%
BTR70	1.00	16.31	98%	93%	BTR70	0.00	ND	0%	0%
SUV	1.00	13.13	100%	63%	SUV	0.00	ND	0%	0%
T72	1.00	31.84	0%	93%	T72	0.00	ND	0%	0%
Truck	1.00	14.38	100%	61%	Truck	0.00	ND	0%	0%
ZSU23-4	1.00	19.00	71%	90%	ZSU23-4	0.00	ND	0%	0%

**Table A2:** Tracking metrics for PCE 25 (optical video). ND means “no detection”.

**1000 m**					**2500 m**				
**Vehicles**	**EinGT**	**CLE**	**DP@20 Pixels**	**% Detections**	**Vehicles**	**EinGT**	**CLE**	**DP@20 Pixels**	**% Detections**
BMP2	0.99	42.03	0%	75%	BMP2	0.00	ND	0%	0%
BRDM2	1.00	20.74	46%	32%	BRDM2	0.00	60.80	0%	2%
BTR70	1.00	30.86	0%	95%	BTR70	0.00	70.78	0%	1%
SUV	1.00	23.91	17%	11%	SUV	0.00	ND	0%	0%
T72	1.00	62.76	0%	89%	T72	0.00	ND	0%	0%
Truck	0.99	27.12	5%	39%	Truck	0.00	ND	0%	0%
ZSU23-4	0.99	36.10	0%	73%	ZSU23-4	0.00	80.06	0%	1%
**1500 m**					**3000 m**				
**Vehicles**	**EinGT**	**CLE**	**DP@20 Pixels**	**% Detections**	**Vehicles**	**EinGT**	**CLE**	**DP@20 Pixels**	**% Detections**
BMP2	1.00	28.66	0%	75%	BMP2	0.67	23.34	67%	1%
BRDM2	1.00	16.18	100%	32%	BRDM2	0.33	48.26	33%	1%
BTR70	1.00	24.27	7%	95%	BTR70	0.97	9.98	98%	28%
SUV	0.00	ND	0%	0%	SUV	0.00	79.13	0%	0%
T72	1.00	50.04	0%	89%	T72	0.88	21.18	75%	2%
Truck	0.00	ND	0%	0%	Truck	0.87	15.27	87%	8%
ZSU23-4	1.00	26.68	6%	73%	ZSU23-4	0.74	16.27	84%	5%
**2000 m**					**3500 m**				
**Vehicles**	**EinGT**	**CLE**	**DP@20 Pixels**	**% Detections**	**Vehicles**	**EinGT**	**CLE**	**DP@20 Pixels**	**% Detections**
BMP2	0.00	ND	0%	36%	BMP2	0.00	ND	0%	0%
BRDM2	0.00	ND	0%	1%	BRDM2	0.00	49.80	19%	4%
BTR70	0.00	ND	0%	38%	BTR70	0.00	37.16	25%	1%
SUV	0.00	ND	0%	0%	SUV	0.00	64.68	0%	2%
T72	0.00	ND	0%	68%	T72	0.00	28.92	33%	1%
Truck	0.00	ND	0%	0%	Truck	0.00	64.58	0%	2%
ZSU23-4	1.00	19.70	100%	47%	ZSU23-4	0.00	ND	0%	0%

## Appendix B. Classification Results for Optical Case: PCE 50 and PCE 25

**Table A3:** Classification results for PCE 50 (optical video) case. Left shows the confusion matrix and the last column shows the classification accuracy.

**1000 m**									**2500 m**								
**Vehicles**	**BMP2**	**BRDM2**	**BTR70**	**SUV**	**T72**	**Truck**	**ZSU23-4**	**Accuracy**	**Vehicles**	**BMP2**	**BRDM2**	**BTR70**	**SUV**	**T72**	**Truck**	**ZSU23-4**	**Accuracy**
BMP2	23	0	71	30	105	13	40	8%	BMP2	0	0	0	0	0	0	0	0%
BRDM2	0	39	1	90	5	142	0	14%	BRDM2	0	0	0	0	0	0	0	0%
BTR70	0	0	244	26	21	20	1	84%	BTR70	0	0	0	0	0	0	0	0%
SUV	0	0	0	120	0	173	0	41%	SUV	0	0	0	0	0	0	0	0%
T72	0	0	7	39	251	35	3	75%	T72	0	0	0	0	0	0	0	0%
Truck	0	3	8	52	13	220	1	75%	Truck	0	0	0	0	0	0	0	0%
ZSU23-4	0	0	0	42	16	21	203	72%	ZSU23-4	0	0	0	0	0	0	0	0%
**1500 m**									**3000 m**								
**Vehicles**	**BMP2**	**BRDM2**	**BTR70**	**SUV**	**T72**	**Truck**	**ZSU23-4**	**Accuracy**	**Vehicles**	**BMP2**	**BRDM2**	**BTR70**	**SUV**	**T72**	**Truck**	**ZSU23-4**	**Accuracy**
BMP2	52	0	99	47	157	5	14	14%	BMP2	0	0	0	0	1	0	0	0%
BRDM2	0	27	36	82	25	173	16	8%	BRDM2	0	0	0	0	0	9	0	0%
BTR70	0	0	344	1	28	0	1	92%	BTR70	0	0	0	0	20	0	0	0%
SUV	0	0	0	126	1	246	0	34%	SUV	0	0	0	0	0	0	0	0%
T72	0	0	36	6	326	5	0	87%	T72	0	0	0	0	2	0	0	100%
Truck	0	4	25	5	45	284	10	76%	Truck	0	0	0	0	4	0	0	0%
ZSU23-4	0	0	26	1	125	7	215	57%	ZSU23-4	0	0	0	0	15	0	0	0%
**2000 m**									**3500 m**								
**Vehicles**	**BMP2**	**BRDM2**	**BTR70**	**SUV**	**T72**	**Truck**	**ZSU23-4**	**Accuracy**	**Vehicles**	**BMP2**	**BRDM2**	**BTR70**	**SUV**	**T72**	**Truck**	**ZSU23-4**	**Accuracy**
BMP2	6	0	105	127	48	69	0	2%	BMP2	0	0	0	0	0	0	0	0%
BRDM2	0	0	3	1	2	13	0	0%	BRDM2	0	0	0	0	0	0	0	0%
BTR70	0	0	293	12	8	36	0	84%	BTR70	0	0	0	0	0	0	0	0%
SUV	0	0	5	23	7	200	0	10%	SUV	0	0	0	0	0	0	0	0%
T72	0	0	12	130	79	127	0	23%	T72	0	0	0	0	0	0	0	0%
Truck	1	1	23	5	42	156	0	68%	Truck	0	0	0	0	0	0	0	0%
ZSU23-4	0	0	87	47	106	93	3	1%	ZSU23-4	0	0	0	0	0	0	0	0%

**Table A4:** Classification results for PCE 25 (optical video) case. Left shows the confusion matrix and the last column shows the classification accuracy.

**1000 m**									**2500 m**								
**Vehicles**	**BMP2**	**BRDM2**	**BTR70**	**SUV**	**T72**	**Truck**	**ZSU23-4**	**Accuracy**	**Vehicles**	**BMP2**	**BRDM2**	**BTR70**	**SUV**	**T72**	**Truck**	**ZSU23-4**	**Accuracy**
BMP2	8	0	47	15	181	11	17	3%	BMP2	0	0	0	0	0	0	0	0%
BRDM2	0	0	1	30	8	78	1	0%	BRDM2	0	0	0	0	9	0	0	0%
BTR70	0	1	179	19	146	9	1	49%	BTR70	0	0	0	1	1	0	0	0%
SUV	0	0	0	20	2	19	0	49%	SUV	0	0	0	0	0	0	0	0%
T72	0	0	12	16	273	29	2	82%	T72	0	0	0	0	0	0	0	0%
Truck	0	1	2	32	19	91	2	62%	Truck	0	0	0	0	1	0	0	0%
ZSU23-4	0	4	12	31	130	21	74	27%	ZSU23-4	0	0	0	0	3	0	0	0%
**1500 m**									**3000 m**								
**Vehicles**	**BMP2**	**BRDM2**	**BTR70**	**SUV**	**T72**	**Truck**	**ZSU23-4**	**Accuracy**	**Vehicles**	**BMP2**	**BRDM2**	**BTR70**	**SUV**	**T72**	**Truck**	**ZSU23-4**	**Accuracy**
BMP2	2	0	46	9	78	0	1	1%	BMP2	0	0	0	0	3	0	0	0%
BRDM2	0	0	0	0	3	1	0	0%	BRDM2	0	0	0	1	2	0	0	0%
BTR70	0	0	112	1	28	0	0	79%	BTR70	0	1	10	0	91	1	2	10%
SUV	0	0	0	0	0	0	0	0%	SUV	0	0	0	0	1	0	0	0%
T72	0	0	16	9	226	2	0	89%	T72	0	0	0	0	8	0	0	100%
Truck	0	0	0	0	0	0	0	0%	Truck	1	0	2	0	28	0	0	0%
ZSU23-4	0	0	15	3	100	1	56	32%	ZSU23-4	0	0	0	1	18	0	0	0%
**2000 m**									**3500 m**								
**Vehicles**	**BMP2**	**BRDM2**	**BTR70**	**SUV**	**T72**	**Truck**	**ZSU23-4**	**Accuracy**	**Vehicles**	**BMP2**	**BRDM2**	**BTR70**	**SUV**	**T72**	**Truck**	**ZSU23-4**	**Accuracy**
BMP2	0	0	0	0	0	0	0	0%	BMP2	0	0	0	0	0	0	0	0%
BRDM2	0	0	0	0	0	0	0	0%	BRDM2	0	0	0	0	16	0	0	0%
BTR70	0	0	0	0	0	0	0	0%	BTR70	0	0	1	0	2	0	1	25%
SUV	0	0	0	0	0	0	0	0%	SUV	0	0	0	0	9	0	0	0%
T72	0	0	0	0	0	0	0	0%	T72	0	0	0	0	3	0	0	100%
Truck	0	0	0	0	0	0	0	0%	Truck	0	0	2	0	5	0	0	0%
ZSU23-4	0	0	0	1	0	0	0	0%	ZSU23-4	0	0	0	0	1	0	0	0%

## Appendix C. Tracking Results for MWIR Daytime Case: PCE 50 and PCE 25

**Table A5:** Tracking metrics for PCE 50 (MWIR daytime) case. 1500 m and 3000 m were used for training.

**1000 m**					**2500 m**				
**Vehicles**	**EinGT**	**CLE**	**DP@20 Pixels**	**% Detections**	**Vehicles**	**EinGT**	**CLE**	**DP@20 Pixels**	**% Detections**
BMP2	0.00	ND	0%	0%	BMP2	1.00	10.30	100%	40%
BRDM2	1.00	26.45	1%	22%	BRDM2	0.00	57.55	0%	20%
BTR70	1.00	26.29	9%	13%	BTR70	0.00	64.52	0%	12%
SUV	1.00	17.08	78%	6%	SUV	0.15	52.81	15%	4%
T72	1.00	34.35	0%	44%	T72	0.00	90.20	0%	0%
Truck	1.00	26.79	33%	1%	Truck	0.00	ND	0%	0%
ZSU23-4	1.00	27.80	2%	37%	ZSU23-4	0.67	32.38	67%	2%
**1500 m**					**3000 m**				
**Vehicles**	**EinGT**	**CLE**	**DP@20 Pixels**	**% Detections**	**Vehicles**	**EinGT**	**CLE**	**DP@20 Pixels**	**% Detections**
BMP2	1.00	22.96	13%	35%	BMP2	0.97	9.11	99%	28%
BRDM2	1.00	19.59	55%	84%	BRDM2	0.92	9.02	95%	55%
BTR70	1.00	18.08	79%	71%	BTR70	0.95	8.38	96%	74%
SUV	0.95	13.30	97%	18%	SUV	0.77	14.09	87%	20%
T72	1.00	25.45	2%	92%	T72	0.99	8.46	100%	75%
Truck	1.00	16.80	81%	28%	Truck	0.85	13.79	87%	36%
ZSU23-4	1.00	29.09	39%	94%	ZSU23-4	0.93	9.39	95%	75%
**2000 m**					**3500 m**				
**Vehicles**	**EinGT**	**CLE**	**DP@20 Pixels**	**% Detections**	**Vehicles**	**EinGT**	**CLE**	**DP@20 Pixels**	**% Detections**
BMP2	1.00	14.96	97%	10%	BMP2	0.00	41.93	0%	1%
BRDM2	1.00	13.34	92%	7%	BRDM2	0.00	65.25	6%	5%
BTR70	0.00	ND	0%	0%	BTR70	0.00	47.56	8%	11%
SUV	0.00	ND	0%	0%	SUV	0.00	40.59	12%	9%
T72	1.00	18.18	84%	16%	T72	0.00	6.23	100%	19%
Truck	0.00	ND	0%	0%	Truck	0.00	45.27	38%	4%
ZSU23-4	1.00	13.09	100%	8%	ZSU23-4	0.00	56.06	13%	26%

**Table A6:** Tracking metrics for PCE 25 (MWIR daytime) case. 1500 m and 3000 m were used for training.

**1000 m**					**2500 m**				
**Vehicles**	**EinGT**	**CLE**	**DP@20 Pixels**	**% Detections**	**Vehicles**	**EinGT**	**CLE**	**DP@20 Pixels**	**% Detections**
BMP2	0.94	33.26	0%	4%	BMP2	0.00	65.38	0%	3%
BRDM2	0.99	29.04	5%	33%	BRDM2	0.03	79.44	5%	11%
BTR70	0.99	25.98	37%	23%	BTR70	0.02	76.91	6%	34%
SUV	0.97	18.41	71%	16%	SUV	0.01	57.69	7%	41%
T72	1.00	35.50	0%	63%	T72	0.17	70.40	18%	58%
Truck	0.93	40.05	27%	4%	Truck	0.16	56.61	25%	18%
ZSU23-4	0.99	30.54	1%	50%	ZSU23-4	0.24	49.33	25%	36%
**1500 m**					**3000 m**				
**Vehicles**	**EinGT**	**CLE**	**DP@20 Pixels**	**% Detections**	**Vehicles**	**EinGT**	**CLE**	**DP@20 Pixels**	**% Detections**
BMP2	0.99	23.28	25%	51%	BMP2	1.00	9.26	100%	1%
BRDM2	1.00	20.59	47%	80%	BRDM2	0.37	37.58	53%	14%
BTR70	0.98	21.89	61%	69%	BTR70	0.52	34.13	61%	17%
SUV	0.90	17.83	87%	18%	SUV	0.23	39.47	45%	9%
T72	0.98	28.99	1%	92%	T72	0.95	9.62	100%	16%
Truck	0.96	22.78	59%	23%	Truck	0.19	34.05	50%	10%
ZSU23-4	1.00	21.65	34%	96%	ZSU23-4	0.55	26.26	65%	18%
**2000 m**					**3500 m**				
**Vehicles**	**EinGT**	**CLE**	**DP@20 Pixels**	**% Detections**	**Vehicles**	**EinGT**	**CLE**	**DP@20 Pixels**	**% Detections**
BMP2	0.95	17.22	88%	11%	BMP2	0.00	38.44	33%	1%
BRDM2	1.00	16.89	85%	4%	BRDM2	0.00	61.67	17%	10%
BTR70	0.25	40.64	25%	1%	BTR70	0.00	53.36	14%	14%
SUV	0.00	ND	0%	0%	SUV	0.00	57.48	7%	11%
T72	0.91	31.45	64%	6%	T72	0.00	96.71	0%	1%
Truck	1.00	13.08	100%	1%	Truck	0.00	53.72	3%	10%
ZSU23-4	0.94	19.68	94%	4%	ZSU23-4	0.00	50.44	19%	22%

## Appendix D. Classification Results for MWIR Daytime Case: PCE 50 and PCE 25

**Table A7:** Classification results for PCE 50 case (MWIR daytime) case. Left shows the confusion matrix and the last column shows the classification accuracy.

**1000 m**									**2500 m**								
**Vehicles**	**BMP2**	**BRDM2**	**BTR70**	**SUV**	**T72**	**Truck**	**ZSU23-4**	**Accuracy**	**Vehicles**	**BMP2**	**BRDM2**	**BTR70**	**SUV**	**T72**	**Truck**	**ZSU23-4**	**Accuracy**
BMP2	0	0	0	0	0	0	0	0%	BMP2	0	0	0	0	1	0	0	0%
BRDM2	2	66	3	2	0	5	0	88%	BRDM2	0	65	4	2	0	0	0	92%
BTR70	5	8	23	9	1	1	0	49%	BTR70	0	33	6	2	2	0	0	14%
SUV	1	0	0	19	2	1	0	83%	SUV	0	7	0	1	3	1	1	8%
T72	61	52	1	8	25	10	0	16%	T72	0	2	1	0	1	1	0	20%
Truck	0	0	0	0	0	3	0	100%	Truck	0	0	0	0	0	0	0	0%
ZSU23-4	2	38	1	8	2	64	18	14%	ZSU23-4	0	4	0	0	2	0	0	0%
**1500 m**									**3000 m**								
**Vehicles**	**BMP2**	**BRDM2**	**BTR70**	**SUV**	**T72**	**Truck**	**ZSU23-4**	**Accuracy**	**Vehicles**	**BMP2**	**BRDM2**	**BTR70**	**SUV**	**T72**	**Truck**	**ZSU23-4**	**Accuracy**
BMP2	4	3	4	5	13	97	1	3%	BMP2	42	11	10	12	12	11	1	42%
BRDM2	0	263	0	2	1	34	0	88%	BRDM2	4	113	1	12	50	8	8	58%
BTR70	1	122	65	4	7	57	0	25%	BTR70	5	132	25	13	47	10	32	9%
SUV	0	11	0	36	3	13	0	57%	SUV	8	9	0	17	23	8	6	24%
T72	4	190	2	4	99	30	0	30%	T72	19	33	1	36	161	14	7	59%
Truck	0	8	0	3	0	89	0	89%	Truck	3	20	2	18	60	15	10	12%
ZSU23-4	0	176	0	7	4	117	32	10%	ZSU23-4	2	166	8	14	29	21	31	11%
**2000 m**									**3500 m**								
**Vehicles**	**BMP2**	**BRDM2**	**BTR70**	**SUV**	**T72**	**Truck**	**ZSU23-4**	**Accuracy**	**Vehicles**	**BMP2**	**BRDM2**	**BTR70**	**SUV**	**T72**	**Truck**	**ZSU23-4**	**Accuracy**
BMP2	1	0	0	4	7	22	2	3%	BMP2	0	1	0	0	0	0	1	0%
BRDM2	0	7	0	0	6	11	1	28%	BRDM2	2	0	1	5	8	1	0	0%
BTR70	0	0	0	0	0	0	0	0%	BTR70	5	1	1	0	30	0	3	3%
SUV	0	0	0	0	0	0	0	0%	SUV	1	1	2	1	24	0	5	3%
T72	0	4	1	4	43	4	0	77%	T72	0	4	1	0	60	0	3	88%
Truck	0	0	0	0	0	0	0	0%	Truck	2	0	0	0	13	0	1	0%
ZSU23-4	0	4	0	0	1	18	6	15%	ZSU23-4	13	29	3	7	37	1	5	5%

**Table A8:** Classification results for PCE 25 (MWIR daytime) case. Left shows the confusion matrix and the last column shows the classification accuracy.

**1000 m**									**2500 m**								
**Vehicles**	**BMP2**	**BRDM2**	**BTR70**	**SUV**	**T72**	**Truck**	**ZSU23-4**	**Accuracy**	**Vehicles**	**BMP2**	**BRDM2**	**BTR70**	**SUV**	**T72**	**Truck**	**ZSU23-4**	**Accuracy**
BMP2	0	3	2	3	4	4	0	0%	BMP2	1	1	1	1	4	1	2	9%
BRDM2	6	89	1	7	1	16	0	74%	BRDM2	0	8	0	4	19	5	2	21%
BTR70	6	18	39	15	3	3	0	46%	BTR70	6	51	10	10	30	11	5	8%
SUV	1	7	0	41	4	6	0	69%	SUV	5	63	7	18	35	13	7	12%
T72	58	47	9	21	51	40	1	22%	T72	7	52	14	13	103	12	7	50%
Truck	1	1	1	0	0	12	0	80%	Truck	6	18	1	8	25	4	2	6%
ZSU23-4	2	47	2	15	2	103	7	4%	ZSU23-4	1	74	5	6	25	11	7	5%
**1500 m**									**3000 m**								
**Vehicles**	**BMP2**	**BRDM2**	**BTR70**	**SUV**	**T72**	**Truck**	**ZSU23-4**	**Accuracy**	**Vehicles**	**BMP2**	**BRDM2**	**BTR70**	**SUV**	**T72**	**Truck**	**ZSU23-4**	**Accuracy**
BMP2	2	21	6	6	25	118	4	1%	BMP2	1	0	0	1	0	0	0	50%
BRDM2	1	133	4	14	6	130	0	46%	BRDM2	8	9	1	6	16	2	7	18%
BTR70	1	70	25	18	7	125	3	10%	BTR70	6	16	3	4	24	4	5	5%
SUV	0	20	0	11	3	28	1	17%	SUV	5	8	1	2	11	3	1	6%
T72	6	135	7	20	79	83	1	25%	T72	2	4	0	0	51	0	1	89%
Truck	0	8	0	1	3	69	2	83%	Truck	5	4	2	2	18	2	3	6%
ZSU23-4	1	115	4	15	9	194	8	2%	ZSU23-4	4	17	2	9	25	4	4	6%
**2000 m**									**3500 m**								
**Vehicles**	**BMP2**	**BRDM2**	**BTR70**	**SUV**	**T72**	**Truck**	**ZSU23-4**	**Accuracy**	**Vehicles**	**BMP2**	**BRDM2**	**BTR70**	**SUV**	**T72**	**Truck**	**ZSU23-4**	**Accuracy**
BMP2	0	1	0	1	8	29	1	0%	BMP2	0	2	0	1	0	0	0	0%
BRDM2	0	2	0	0	5	6	0	15%	BRDM2	4	3	3	3	20	0	2	9%
BTR70	0	0	0	2	0	2	0	0%	BTR70	3	1	0	2	41	1	1	0%
SUV	0	0	0	0	0	0	0	0%	SUV	6	4	1	3	19	0	8	7%
T72	0	2	0	2	13	5	0	59%	T72	0	0	0	2	0	0	0	0%
Truck	0	0	0	0	4	1	0	20%	Truck	7	4	1	4	16	2	1	6%
ZSU23-4	0	1	0	2	4	9	0	0%	ZSU23-4	10	19	3	10	28	7	3	4%

## Appendix E. Tracking Results for MWIR Nighttime Case: PCE 50 and PCE 25

**Table A9:** Tracking metrics for PCE 50 (MWIR nighttime). ND means no detection.

**1000 m**					**2500 m**				
**Vehicles**	**EinGT**	**CLE**	**DP@20 Pixels**	**% Detections**	**Vehicles**	**EinGT**	**CLE**	**DP@20 Pixels**	**% Detections**
BMP2	1.00	29.11	4%	45%	BMP2	0.00	ND	0%	0%
BRDM2	1.00	24.23	27%	87%	BRDM2	0.00	ND	0%	0%
BTR70	1.00	15.42	85%	86%	BTR70	0.00	ND	0%	0%
SUV	1.00	13.93	100%	96%	SUV	0.00	ND	0%	0%
T72	1.00	31.04	0%	82%	T72	0.00	ND	0%	0%
Truck	1.00	26.22	0%	56%	Truck	0.00	ND	0%	0%
ZSU23-4	1.00	25.39	6%	86%	ZSU23-4	0.00	ND	0%	0%
**1500 m**					**3000 m**				
**Vehicles**	**EinGT**	**CLE**	**DP@20 Pixels**	**% Detections**	**Vehicles**	**EinGT**	**CLE**	**DP@20 Pixels**	**% Detections**
BMP2	1.00	19.67	53%	95%	BMP2	0.00	ND	0%	0%
BRDM2	1.00	19.89	52%	85%	BRDM2	0.00	ND	0%	0%
BTR70	1.00	12.17	100%	94%	BTR70	0.00	ND	0%	0%
SUV	1.00	10.18	100%	93%	SUV	0.00	ND	0%	0%
T72	1.00	23.98	3%	96%	T72	0.00	ND	0%	0%
Truck	1.00	18.95	73%	95%	Truck	0.00	ND	0%	0%
ZSU23-4	1.00	20.24	53%	95%	ZSU23-4	0.00	ND	0%	0%
**2000 m**					**3500 m**				
**Vehicles**	**EinGT**	**CLE**	**DP@20 Pixels**	**% Detections**	**Vehicles**	**EinGT**	**CLE**	**DP@20 Pixels**	**% Detections**
BMP2	1.00	15.77	91%	3%	BMP2	0.00	ND	0%	0%
BRDM2	0.99	14.38	97%	75%	BRDM2	0.00	ND	0%	0%
BTR70	1.00	7.76	100%	48%	BTR70	0.00	ND	0%	0%
SUV	1.00	6.56	100%	86%	SUV	0.00	ND	0%	0%
T72	1.00	16.62	97%	78%	T72	0.00	ND	0%	0%
Truck	1.00	14.40	95%	22%	Truck	0.00	ND	0%	0%
ZSU23-4	1.00	13.67	100%	63%	ZSU23-4	0.00	ND	0%	0%

**Table A10:** Tracking metrics for PCE 25 (MWIR nighttime). ND means no detection.

**1000 m**					**2500 m**				
**Vehicles**	**EinGT**	**CLE**	**DP@20 Pixels**	**% Detections**	**Vehicles**	**EinGT**	**CLE**	**DP@20 Pixels**	**% Detections**
BMP2	1.00	27.93	0%	72%	BMP2	0.00	ND	0%	0%
BRDM2	1.00	25.71	4%	100%	BRDM2	0.00	ND	0%	0%
BTR70	1.00	15.87	83%	100%	BTR70	0.00	ND	0%	0%
SUV	1.00	13.58	100%	100%	SUV	0.00	ND	0%	0%
T72	1.00	31.81	0%	99%	T72	0.00	ND	0%	0%
Truck	1.00	26.32	0%	100%	Truck	0.00	ND	0%	0%
ZSU23-4	1.00	25.95	0%	100%	ZSU23-4	0.00	ND	0%	0%
**1500 m**					**3000 m**				
**Vehicles**	**EinGT**	**CLE**	**DP@20 Pixels**	**% Detections**	**Vehicles**	**EinGT**	**CLE**	**DP@20 Pixels**	**% Detections**
BMP2	1.00	19.26	60%	100%	BMP2	0.00	ND	0%	0%
BRDM2	1.00	18.83	73%	100%	BRDM2	0.00	ND	0%	0%
BTR70	1.00	11.41	100%	100%	BTR70	0.00	ND	0%	0%
SUV	1.00	10.08	100%	100%	SUV	0.00	ND	0%	0%
T72	1.00	23.10	10%	100%	T72	0.00	ND	0%	0%
Truck	1.00	19.05	69%	99%	Truck	0.00	ND	0%	0%
ZSU23-4	1.00	18.97	75%	100%	ZSU23-4	0.00	ND	0%	0%
**2000 m**					**3500 m**				
**Vehicles**	**EinGT**	**CLE**	**DP@20 Pixels**	**% Detections**	**Vehicles**	**EinGT**	**CLE**	**DP@20 Pixels**	**% Detections**
BMP2	1.00	13.77	99%	23%	BMP2	0.00	ND	0%	0%
BRDM2	1.00	13.65	99%	96%	BRDM2	0.00	ND	0%	0%
BTR70	1.00	7.75	100%	68%	BTR70	0.00	ND	0%	0%
SUV	1.00	7.22	100%	58%	SUV	0.00	ND	0%	0%
T72	1.00	16.67	97%	95%	T72	0.00	ND	0%	0%
Truck	1.00	15.11	94%	23%	Truck	0.00	ND	0%	0%
ZSU23-4	1.00	13.88	99%	79%	ZSU23-4	0.00	ND	0%	0%

## Appendix F. Classification Results for MWIR Nighttime Case: PCE 50 and PCE 25

**Table A11:** Classification results for PCE 50 case (MWIR nighttime). Left shows the confusion matrix and the last column shows the classification accuracy.

**1000 m**									**2500 m**								
**Vehicles**	**BMP2**	**BRDM2**	**BTR70**	**SUV**	**T72**	**Truck**	**ZSU23-4**	**Accuracy**	**Vehicles**	**BMP2**	**BRDM2**	**BTR70**	**SUV**	**T72**	**Truck**	**ZSU23-4**	**Accuracy**
BMP2	110	29	0	1	19	0	1	69%	BMP2	0	0	0	0	0	0	0	0%
BRDM2	0	256	0	12	11	0	33	80%	BRDM2	0	0	0	0	0	0	0	0%
BTR70	16	115	128	0	36	1	14	41%	BTR70	0	0	0	0	0	0	0	0%
SUV	0	79	0	241	10	15	0	70%	SUV	0	0	0	0	0	0	0	0%
T72	3	46	1	2	228	2	12	78%	T72	0	0	0	0	0	0	0	0%
Truck	0	51	0	0	8	141	0	71%	Truck	0	0	0	0	0	0	0	0%
ZSU23-4	3	58	2	2	54	0	190	61%	ZSU23-4	0	0	0	0	0	0	0	0%
**1500 m**									**3000 m**								
**Vehicles**	**BMP2**	**BRDM2**	**BTR70**	**SUV**	**T72**	**Truck**	**ZSU23-4**	**Accuracy**	**Vehicles**	**BMP2**	**BRDM2**	**BTR70**	**SUV**	**T72**	**Truck**	**ZSU23-4**	**Accuracy**
BMP2	136	85	0	1	87	5	26	40%	BMP2	0	0	0	0	0	0	0	0%
BRDM2	0	301	0	0	3	0	0	99%	BRDM2	0	0	0	0	0	0	0	0%
BTR70	0	116	204	0	18	0	0	60%	BTR70	0	0	0	0	0	0	0	0%
SUV	0	66	0	187	9	69	2	56%	SUV	0	0	0	0	0	0	0	0%
T72	2	16	0	0	324	3	1	94%	T72	0	0	0	0	0	0	0	0%
Truck	2	100	4	1	62	171	1	50%	Truck	0	0	0	0	0	0	0	0%
ZSU23-4	0	33	0	0	69	7	233	68%	ZSU23-4	0	0	0	0	0	0	0	0%
**2000 m**									**3500 m**								
**Vehicles**	**BMP2**	**BRDM2**	**BTR70**	**SUV**	**T72**	**Truck**	**ZSU23-4**	**Accuracy**	**Vehicles**	**BMP2**	**BRDM2**	**BTR70**	**SUV**	**T72**	**Truck**	**ZSU23-4**	**Accuracy**
BMP2	0	7	0	0	4	0	0	0%	BMP2	0	0	0	0	0	0	0	0%
BRDM2	13	197	0	1	30	2	25	74%	BRDM2	0	0	0	0	0	0	0	0%
BTR70	0	64	55	0	46	4	4	32%	BTR70	0	0	0	0	0	0	0	0%
SUV	0	56	25	59	84	82	1	19%	SUV	0	0	0	0	0	0	0	0%
T72	2	2	2	0	253	13	7	91%	T72	0	0	0	0	0	0	0	0%
Truck	0	19	9	0	20	29	3	36%	Truck	0	0	0	0	0	0	0	0%
ZSU23-4	0	38	3	3	65	35	83	37%	ZSU23-4	0	0	0	0	0	0	0	0%

**Table A12:** Classification results for PCE 25 case (MWIR nighttime). Left shows the confusion matrix and the last column shows the classification accuracy.

**1000 m**									**2500 m**								
**Vehicles**	**BMP2**	**BRDM2**	**BTR70**	**SUV**	**T72**	**Truck**	**ZSU23-4**	**Accuracy**	**Vehicles**	**BMP2**	**BRDM2**	**BTR70**	**SUV**	**T72**	**Truck**	**ZSU23-4**	**Accuracy**
BMP2	198	14	0	0	45	0	2	76%	BMP2	0	0	0	0	0	0	0	0%
BRDM2	0	357	0	0	2	0	0	99%	BRDM2	0	0	0	0	0	0	0	0%
BTR70	13	196	95	0	53	0	2	26%	BTR70	0	0	0	0	0	0	0	0%
SUV	1	126	0	135	36	61	0	38%	SUV	0	0	0	0	0	0	0	0%
T72	2	57	0	0	288	6	4	81%	T72	0	0	0	0	0	0	0	0%
Truck	1	89	1	0	60	207	1	58%	Truck	0	0	0	0	0	0	0	0%
ZSU23-4	1	66	1	0	38	2	251	70%	ZSU23-4	0	0	0	0	0	0	0	0%
**1500 m**									**3000 m**								
**Vehicles**	**BMP2**	**BRDM2**	**BTR70**	**SUV**	**T72**	**Truck**	**ZSU23-4**	**Accuracy**	**Vehicles**	**BMP2**	**BRDM2**	**BTR70**	**SUV**	**T72**	**Truck**	**ZSU23-4**	**Accuracy**
BMP2	59	170	3	0	99	15	12	16%	BMP2	0	0	0	0	0	0	0	0%
BRDM2	0	344	0	0	14	1	0	96%	BRDM2	0	0	0	0	0	0	0	0%
BTR70	0	181	117	0	56	1	4	33%	BTR70	0	0	0	0	0	0	0	0%
SUV	0	88	2	70	84	112	3	19%	SUV	0	0	0	0	0	0	0	0%
T72	0	16	0	0	329	13	1	92%	T72	0	0	0	0	0	0	0	0%
Truck	1	105	7	1	125	115	1	32%	Truck	0	0	0	0	0	0	0	0%
ZSU23-4	0	46	4	0	124	8	176	49%	ZSU23-4	0	0	0	0	0	0	0	0%
**2000 m**									**3500 m**								
**Vehicles**	**BMP2**	**BRDM2**	**BTR70**	**SUV**	**T72**	**Truck**	**ZSU23-4**	**Accuracy**	**Vehicles**	**BMP2**	**BRDM2**	**BTR70**	**SUV**	**T72**	**Truck**	**ZSU23-4**	**Accuracy**
BMP2	13	47	0	0	28	6	0	16%	BMP2	0	0	0	0	0	0	0	0%
BRDM2	0	279	2	1	59	2	0	81%	BRDM2	0	0	0	0	0	0	0	0%
BTR70	0	100	49	0	88	5	1	20%	BTR70	0	0	0	0	0	0	0	0%
SUV	0	41	10	12	98	49	0	6%	SUV	0	0	0	0	0	0	0	0%
T72	2	16	0	2	280	38	3	82%	T72	0	0	0	0	0	0	0	0%
Truck	0	13	0	1	50	20	0	24%	Truck	0	0	0	0	0	0	0	0%
ZSU23-4	0	63	11	3	114	32	60	21%	ZSU23-4	0	0	0	0	0	0	0	0%

## References

[1] Candes E.J., Wakin M.B. (2008). An introduction to compressive sampling. IEEE Signal Process. Mag..

[2] Zhang J., Xiong T., Tran T., Chin S., Etienne-Cummings R. (2016). Compact all-CMOS spatio-temporal compressive sensing video camera with pixel-wise coded exposure. Opt. Express.

[3] Yang J., Zhang Y. (2011). Alternating direction algorithms for l1-problems in compressive sensing. SIAM J. Sci. Comput..

[4] Tropp J.A. (2004). Greed is good: Algorithmic results for sparse approximation. IEEE Trans. Inf. Theory.

[5] Dao M., Kwan C., Koperski K., Marchisio G. A joint sparsity approach to tunnel activity monitoring using high resolution satellite images. Proceedings of the IEEE Ubiquitous Computing, Electronics & Mobile Communication Conference.

[6] Zhou J., Ayhan B., Kwan C., Tran T. (2018). ATR performance improvement using images with corrupted or missing pixels. Pattern Recognition and Tracking XXIX.

[7] Applied Research LLC (2016). Phase 1 Final Report.

[8] Yang M.H., Zhang K., Zhang L. (2012). Real-Time compressive tracking. European Conference on Computer Vision.

[9] Redmon J., Farhadi A. YOLOv3: An Incremental Improvement. https://arxiv.org/abs/1804.02767.

[10] Kwan C., Chou B., Yang J., Rangamani A., Tran T., Zhang J., Etienne-Cummings R. (2019). Target tracking and classification directly using compressive sensing camera for SWIR videos. J. Signal Image Video Process..

[11] Kwan C., Chou B., Yang J., Rangamani A., Tran T., Zhang J., Etienne-Cummings R. (2019). Target tracking and classification using compressive measurements of MWIR and LWIR coded aperture cameras. J. Signal Inf. Process..

[12] Kwan C., Chou B., Yang J., Tran T. (2019). Compressive object tracking and classification using deep learning for infrared videos. Pattern Recognition and Tracking XXX (Conference SI120).

[13] Kwan C., Chou B., Yang J., Tran T. (2019). Target tracking and classification directly in compressive measurement domain for low quality videos. Pattern Recognition and Tracking XXX (Conference SI120).

[14] Kwan C., Chou B., Echavarren A., Budavari B., Li J., Tran T. Compressive vehicle tracking using deep learning. Proceedings of the IEEE Ubiquitous Computing, Electronics & Mobile Communication Conference.

[15] He K., Zhang X., Ren S., Sun J. Deep Residual Learning for Image Recognition. Proceedings of the Conference on Computer Vision and Pattern Recognition.

[16] Bertinetto L., Valmadre J., Golodetz S., Miksik O., Torr P.H. Staple: Complementary learners for real-time tracking. Proceedings of the Conference on Computer Vision and Pattern Recognition.

[17] Stauffer C., Grimson W.E.L. (1999). Adaptive background mixture models for real-time tracking, computer vision and pattern recognition. IEEE Comput. Soc. Conf..

[18] Kulkarni K., Turaga P.K. (2016). Reconstruction-free action inference from compressive imagers. IEEE Trans. Pattern Anal. Mach. Intell..

[19] Lohit S., Kulkarni K., Turaga P.K. Direct inference on compressive measurements using convolutional neural networks. Proceedings of the 2016 IEEE International Conference on Image Processing (ICIP).

[20] Adler A., Elad M., Zibulevsky M. (2016). Compressed Learning: A Deep Neural Network Approach. arXiv.

[21] Xu Y., Kelly K.F. (2019). Compressed Domain Image Classification Using a Multi-Rate Neural Network. arXiv.

[22] Kulkarni K., Turaga P.K. (2016). Fast Integral Image Estimation at 1% Measurement Rate. arXiv.

[23] Wang Z.W., Vineet V., Pittaluga F., Sinha S.N., Cossairt O., Kang S.B. Privacy-preserving action recognition using coded aperture videos. Proceedings of the IEEE Conference on Computer Vision and Pattern Recognition (CVPR) Workshops.

[24] Vargas H., Fonseca Y., Arguello H. Object detection on compressive measurements using correlation filters and sparse representation. Proceedings of the 2018 26th European Signal Processing Conference (EUSIPCO).

[25] Değerli A., Aslan S., Yamac M., Sankur B., Gabbouj M. Compressively sensed image recognition. Proceedings of the 7th European Workshop on Visual Information Processing (EUVIP).

[26] Latorre-Carmona P., Traver V.J., Sánchez J.S., Tajahuerce E. (2019). Online reconstruction-free single-pixel image classification. Image Vis. Comput..

[27] Ren S., He K., Girshick R., Sun J. (2015). Faster R-CNN: Towards real-time object detection with region proposal networks. Advances in Neural Information Processing Systems.

[28] MOT Challenge. https://motchallenge.net/.

